# Through‐Space Polar‐π Interactions in 2,6‐Diarylthiophenols

**DOI:** 10.1002/cphc.202000132

**Published:** 2020-05-08

**Authors:** Jie Jian, Jordi Poater, Roel Hammink, Paul Tinnemans, Christine J. McKenzie, F. Matthias Bickelhaupt, Jasmin Mecinović

**Affiliations:** ^1^ Department of Physics, Chemistry and Pharmacy University of Southern Denmark Campusvej 55 5230 Odense Denmark; ^2^ ICREA Passeig Lluís Companys 23 08010 Barcelona Spain; ^3^ Departament de Química Inorgànica i Orgànica & IQTCUB Universitat de Barcelona Martí i Franquès 1–11 08028 Barcelona Spain; ^4^ Division of Immunotherapy, Oncode Institute Radboud University Medical Center Nijmegen The Netherlands; ^5^ Department of Tumor Immunology Radboud Institute for Molecular Life Sciences Radboud University Medical Center Geert Grooteplein 26 6525 GA Nijmegen The Netherlands; ^6^ Institute for Molecules and Materials Radboud University Heyendaalseweg 135 6525 AJ Nijmegen The Netherlands; ^7^ Department of Theoretical Chemistry Amsterdam Center for Multiscale Modeling Vrije Universiteit Amsterdam De Boelelaan 1083 1081 HV Amsterdam The Netherlands

**Keywords:** aromatic compounds, molecular recognition, noncovalent interactions, polar-π interactions, thiols

## Abstract

Molecular recognition between polar groups and aromatic molecules is fundamentally important to rational drug design. Although it has been well established that many polar functionalities interact with electron‐rich aromatic residues through energetically favorable polar‐π interactions, there is a limited understanding of the association between thiols and aromatic systems. Herein we report physical‐organic chemistry studies on 2,6‐diarylthiophenols that possess the central thiophenol ring and two flanking aromatic rings with tunable electronic properties caused by substituents at distant *para* position. Hammett analysis revealed that p*K*_a_ values and proton affinities correlate well with Hammett sigma values of substituents. Additional energy decomposition analysis supported the conclusion that both through‐space SH‐π interactions and S^−^‐π interactions contribute to intramolecular stabilization of 2,6‐diarylthiophenols.

## Graphical abstract

**Understanding interactions**: Despite a well‐established knowledge that polar‐π interactions play an important role in molecular recognition, it is currently poorly understood how thiols interact with aromatic systems. Our physical‐organic chemistry studies, based on measurements of p*K*_a_ values, structural studies, and quantum chemical analyses, reveal that 2,6‐diarylthiophenols are stabilized by through‐space SH‐π interactions and S^−^‐π interactions.

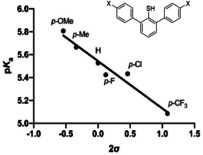

## 1 Introduction

Thiols represent an important class of molecules that play essential roles in molecular and biological systems.^1^, ^2^ The nucleophilic sulfhydryl group (−SH) typically reacts with various electrophiles, thus enabling a preparation of well‐defined biomolecules and biomaterials with novel structure and function.^3^, ^4^ Cysteine, the only thiol in the panel of 22 proteinogenic amino acids, has commonly been used for site‐specific chemoselective modification of peptides and proteins.^5^ Although the chemical reactivity of cysteine and other thiols has been extensively studied,^3^, ^4^, ^5^ the involvement of thiols in molecular recognition has been less understood.^2^, ^6^ The polar SH group, for example, can act both as a hydrogen bond donor and acceptor in the presence of the amide backbone in proteins and small molecules.^7^ Structural analyses of proteins demonstrated that cysteine can form three type of interactions with aromatic rings, namely SH‐π interactions (i. e. interactions between H and the π face), HS‐π interactions (i.e interactions between S and the π face), and HS−HC interactions (i. e. interactions between S and the HC face of the aromatic ring), with the second one being the most common.^2^, ^8^ Computational analyses, however, showed that energetically favorable SH‐π interactions with the π system of aromatic rings appear to be preferred.^9^ In comparison with other types of polar‐π interactions (e. g. OH‐π, NH‐π, cation‐π and π–π interactions), direct SH‐π interactions appear to be less studied and established.^2^

**Figure 1:**
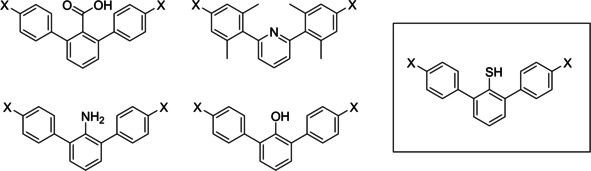
2,6‐Diaryl aromatic systems for examinations of through‐space polar‐π interactions.

Simple small molecular systems have emerged as good models for studies of through‐space polar‐π interactions. For instance, substituted 2,6‐diaryl aromatic systems enabled detailed physical‐organic investigations on carboxylic acids, pyridines, anilines and phenols (Figure 1).^10^, ^11^, ^12^, ^13^ The molecular architecture of the 2,6‐diaryl aromatic system is particularly suitable for examinations of intramolecular polar‐π interactions between a polar group located at the central aromatic ring and the two flanking aromatic rings whose electronic properties can be fine‐tuned by substituents at the distant *para* position. Measurements of p*K*_a_ values, structural analyses, and computational studies revealed the presence of COOH‐π interactions in 2,6‐diarylcarboxylic acids,^11^ NH‐π interactions in 2,6‐diarylpyridines,^12^ cation‐π interactions in 2,6‐diarylanilines,^13^ and OH‐π interactions in 2,6‐diarylphenols.^10^ Inspired by these precedents, here we report experimental and computational investigations of through‐space SH‐π interactions in 2,6‐diarylthiophenols. We hypothesized that there is a linear correlation between the p*K*_a_ values and Hammett σ values in *para*‐substituted 2,6‐diarylthiophenols, leading to stabilization by through‐space, and not by through‐bond, polar‐π interactions.

## 2 Results and Discussion

**Scheme 1:**
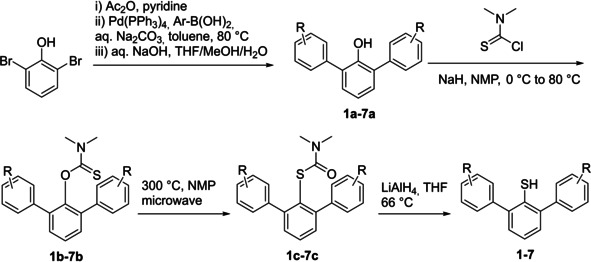
Synthesis of 2,6‐Diarylthiophenols **1**–**7**.

Substituted 2,6‐diarylthiophenols **1**–**7** were synthesized in several steps from 2,6‐dibromophenol (Scheme 1). Based on our previous protocol,^10^ 2,6‐diarylphenols **1 a**–**7 a** were prepared through palladium‐catalyzed Suzuki cross‐coupling between 2,6‐dibromophenol and *para*/*meta*‐substituted phenylboronic acids in good yields. The resulting 2,6‐diarylphenols **1 a**–**7 a** reacted with dimethylthiocarbamoyl chloride in the presence of NaH in N‐methyl pyrrolidone (NMP) to give *O*‐thiocarbamates **1 b**–**7 b**, which underwent the microwave‐mediated Newman‐Kwart rearrangement at 300 °C to produce corresponding *S*‐carbamates **1 c**–**7 c**. Reduction of *S*‐carbamates **1 c**–**7 c** by lithium aluminum hydride (LiAlH_4_) refluxed in tetrahydrofuran (THF) afforded 2,6‐diarylthiophenols **1**–**7**.

**Figure 2:**
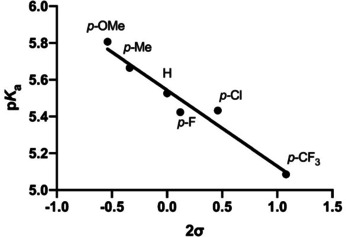
Correlation between p*K*_a_ values of thiophenols **1**–**6** and the Hammett sigma values (2σ).

The acidity of 2,6‐diarylthiophenols **1**–**7** in aqueous solutions (with 10 % DMSO to obtain fully soluble compounds) was measured by UV‐Vis spectroscopy in the range of pH 3–12, similarly as used previously for related 2,6‐diarylphenols (Table 1 and Figure S1 in the Supporting Information).^10^ The experimentally obtained p*K*_a_ values for **1**–**6** were then plotted against the Hammett sigma values for *para*‐substituents (2σ was used because two flanking rings are present) (Figure 2). We observed a strong linear correlation with R^2^=0.95, and the ρ value of +0.42. These results indicate that the acidity constant for 2,6‐diarylthiophenols is affected by the presence of substituents at distant *para* position of the flanking aromatic rings; electron‐donating substituents (e. g. OMe) make thiols weaker acids, whereas electron‐withdrawing substituents (e. g. CF_3_) make thiols stronger acids. The *meta*‐substituted F, having a significantly different σ value than the *para*‐substituted F (0.34 vs. 0.06), was found to have quite similar p*K*_a_ value to its *para* analog. Through‐bond effects appear to play a minor role in the acidity of 2,6‐diarylthiophenols; the inductive effect diminishes with the number of bonds (there are five C−C bonds between the SH and *para*‐X), whereas the resonance effect is excluded due to the nonplanarity of the entire system. Collectively, these results suggest that 2,6‐diarylthiophenols could not be stabilized *via* through‐bond interactions (through resonance and/or inductive effects), but *via* through‐space polar‐π interactions.

**Table 1:** p*K*_a_ Values for Thiophenols **1**–**7**.

Compound	X	*σ*	p*K*_a_^[a]^
**1**	H	0.00	5.52
**2**	*p*‐OMe	−0.27	5.81
**3**	*p*‐Me	−0.17	5.66
**4**	*p*‐F	0.06	5.44
**5**	*p*‐Cl	0.46	5.45
**6**	*p*‐CF_3_	0.54	5.07
**7**	*m*‐F	0.34	5.30

[a] Determined in H_2_O:DMSO=9 : 1.

**Figure 3:**
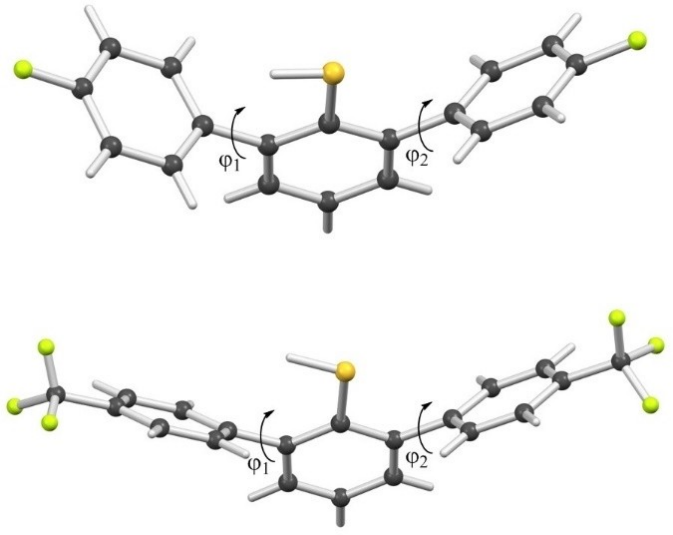
X‐ray crystal structures of **4** (top) and **6** (bottom).

To provide a structural insight into the position of the SH group and the neighboring aromatic rings, we then determined X‐ray structures of thiols **4** and **6** (Figure 3, Figures S2–S6, Tables S1–S5). The X‐ray crystal structure of *para*‐F possessing 2,6‐diphenylthiophenol **4** shows that the three aromatic rings in **4** are not coplanar (Figure 3). The flanking rings are 26° from eclipsing each other when viewed parallel to the central ring (Figure S2). Dihedral angles between the mean planes of the central thiophenol ring and the *ortho para‐*fluorobenzene rings are 89.5° (φ_1_) and 63.7° (φ_2_). The presence of *ortho* aryl groups apparently prevents association between adjacent molecules which, by comparison, are otherwise orientated in head‐to‐head dimers through a combination of H bonding and thiophilic interactions in the X‐ray crystal structure of unsubstituted thiophenol. This arrangement allows for the emergence of the SH‐π interaction between the neighboring ring and the thiophenol moiety in a close to perpendicular orientation. The thiol H atom, which was located in the electron density map, is associated with this aromatic ring with distances of SH−C_α_ 2.4 Å, SH−C_β_ 2.9 Å, SH−C_γ_ 3.6 Å and SH−C_δ_ 3.9 Å (Figure S3). Similarly, the structure elucidation of *para*‐CF_3_ containing 2,6‐diphenylthiophenol **6** showed that the dihedral angles between the central thiophenol ring and the adjacent aromatic rings were 52.0° (φ_1_) and 58.1° (φ_2_), and that the shortest distances between the hydrogen of SH and carbon of flanking rings were SH−C_α_ 2.6 Å, SH−C_β_ 2.5 Å, SH−C_γ_ 3.4 Å and SH−C_δ_ 4.1 Å.

Following our previous computational analyses that constituted an essential part in physical‐organic studies on 2,6‐diarylanilines,^13^ 2,6‐diarylpyridines,^12^ and 2,6‐diarylphenols,^10^ we carried out detailed quantum chemical analyses on the role of through‐space polar‐π interactions in 2,6‐diarylthiophenols. Our objectives were to examine: (1) dependence of the *para*/*meta* substituent on the energy (enthalpy) change for ArSH→ArS^−^+H^+^; (2) for X = H, rotational barrier for internal rotation around the C−C bond linking the central thiophenol ring and the adjacent flanking aromatic rings (i. e. the lowest energy of X = H is computed, followed by calculation of the rotational barrier around C−C for one ring while keeping the other at the lowest energy conformation); and (3) the foundation of the *para*‐substituent effect by means of an energy decomposition analysis. This computational analysis was done using the ADF program at the BLYP‐D3BJ/TZ2P level of dispersion‐corrected DFT in aqueous solvation simulated using COSMO (Tables S6–S13).^14^

**Figure 4:**
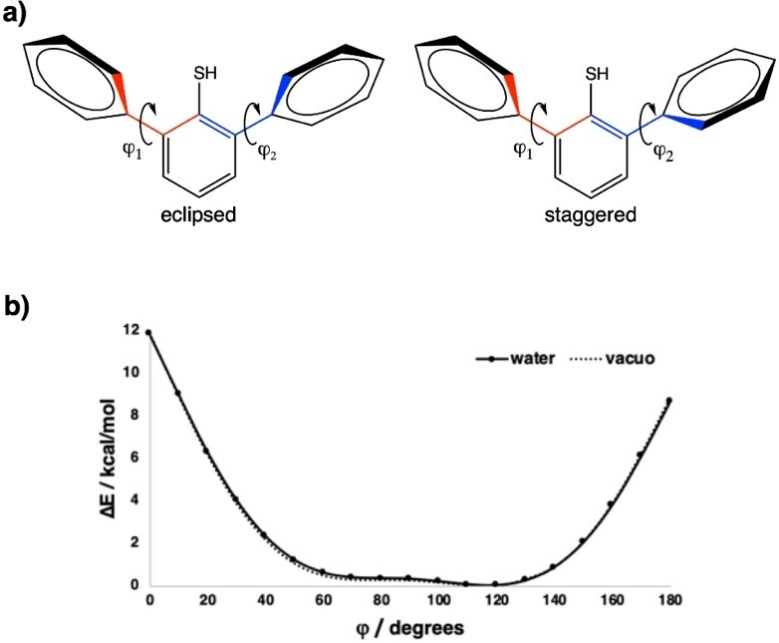
a) Structures of eclipsed and staggered conformations for 2,6‐diarylthiophenol **1**. b) Dependence of the relative energy ▵*E* (in kcal mol^−1^) of 2,6‐diarylthiophenol **1** with the rotation of the HSC−C_2_−C_α_−C_β_ dihedral angle (ϕ, in degrees) in water and in vacuo.

The optimization of the 2,6‐diarylthiophenols **1**–**7** drives to two almost isoenergetic conformations with respect to the dihedral angle with the two aryl rings: eclipsed (also described as parallel) and staggered (also described as antiparallel) (Figure 4a). In agreement with structural analyses (see above), the eclipsed conformation is slightly more stable than the staggered conformation, both in the gas phase and in water, by up to 0.41 kcal mol^−1^ (Table 2 and Tables S14 and S15). In addition, the rotational barrier was calculated for 2,6‐diarylthiophenol (**1**, X = H) in the range 0—180° (Figure 4b). Only the HSC−C_2_−C_α_−C_ß_ dihedral angle was varied, whereas the other angle was kept at either 117.5° (water) or 116.9° (gas). The conformers in the range 50–150° differ by less than 2 kcal mol^−1^, thus confirming again the almost isoenergetic eclipsed and staggered conformations, whereas those close to either 0° or 180° appear to be energetically unfavorable.

**Table 2:** Energy difference between eclipsed and staggered conformations and proton affinities for both conformations in water and in vacuo of the series of 2,6‐diarylthiophenols **1**–**7** (values in kcal mol^−1^).

		Water			Vacuo		
Compound	X	▵▵E_ecli‐stag_	▵E^PA^_ecli_	▵E^PA^_stag_	▵▵E_ecli‐stag_	▵E^PA^_ecli_	▵E^PA^_stag_
**1**	H	−0.30	174.6	174.6	−0.26	341.5	341.3
**2**	*p*‐OMe	−0.41	175.8	175.6	−0.36	343.1	342.8
**3**	*p*‐Me	−0.36	175.3	175.2	−0.31	342.7	342.4
**4**	*p*‐F	−0.32	174.2	174.2	−0.28	336.4	336.1
**5**	*p*‐Cl	−0.35	174.0	174.0	−0.29	334.4	334.1
**6**	*p*‐CF_3_	−0.38	173.3	173.2	−0.27	328.9	328.9
**7**	*m*‐F	−0.25	173.6	173.7	−0.15	336.3	336.3

**Figure 5:**
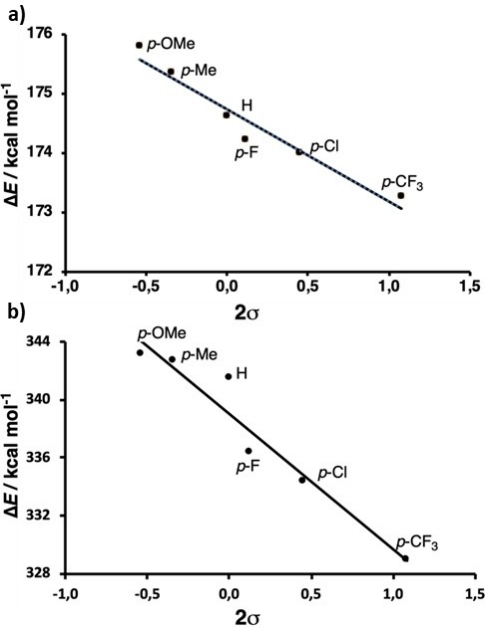
Dependence of calculated proton affinities ▵*E* on the Hammett sigma values (2σ) of 2,6‐diarylthiophenols **1**–**6** in an eclipsed conformation in a) water and b) gas phase.

Table 2 also contains the calculated proton affinities (▵*E* ^PA^) of the set of substituted 2,6‐diarylthiophenols **1**–**7**. Both conformations appear to have indistinguishable ▵*E* ^PA^. Next, we have plotted the ▵*E* ^PA^ for eclipsed conformations against twice the Hammett constant (2σ), and it can be observed that good correlations are obtained both in water (slope=−1.55, R^2^=0.95) and in the gas phase (slope=−9.36, R^2^=0.94) (Figure 5 and Figure S7). The larger slope in the gas phase correlates with the larger ▵*E* ^PA^ in the gas phase than in water. The reduced ▵*E* ^PA^ in aqueous solution is due to the much stronger solvation of the proton than of the protonated thiophenol in which the net positive charge is distributed over a larger volume. In addition, the fact that *meta*‐ and *para*‐substituted F have similar ▵*E* ^PA^ further supports the presence of through‐space interaction in the series of 2,6‐diarylthiophenols.

**Figure 6:**
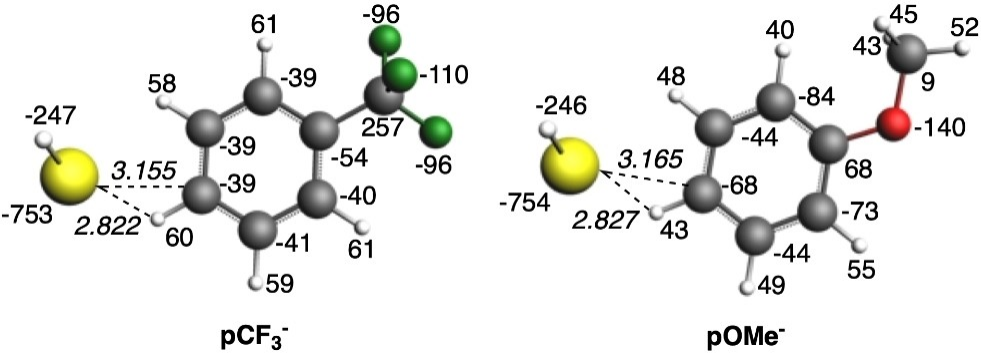
VDD charges (in milli‐electrons) for fragments in model systems used in through‐space interaction analysis for *p*‐CF_3_^−^ and *p*‐OMe^−^. Shortest S⋅⋅⋅C and S⋅⋅⋅H bond lengths (in Å) are also included for both systems (values in italics).

Next, further insight into the molecular origin of the *para*‐substituent effect was obtained by means of an energy decomposition analysis (EDA) on all seven systems, and particularly on **2** (▵*E* ^PA^_ecli_=175.8 kcal mol^−1^) and **6** (▵*E* ^PA^_ecli_=173.3 kcal mol^−1^), which present the largest and the smallest PA, respectively (Table 3). In particular, the EDA has been performed on the interaction between one aryl and either H_2_S (to mimic thiol) or HS^−^ (to mimic thiolate) (results in Table 3, and methodology in Experimental Section).^10^, ^13^ From the interaction energies of *p*‐OMe (−0.6 kcal mol^−1^) and *p*‐CF_3_ (−0.3 kcal mol^−1^), it is evident that SH‐π interactions are quite weak. In addition, their close ▵*E*_int_ values cannot justify the difference in PA values. This is at variance with the unprotonated systems, for which the ▵*E*_int_ for *p*‐CF_3_^−^ is clearly more attractive (−9.7 kcal mol^−1^) than for *p*‐OMe^−^ (−2.0 kcal mol^−1^). The EDA proves that this difference is a result of the reduction of electrostatic interaction from *p*‐CF_3_^−^ to *p*‐OMe^−^ from −9.5 to −3.0 kcal mol^−1^, respectively, whereas ▵*E*_Pauli_, ▵*E*_oi_ and ▵*E*_disp_ are very similar for both substituents (Table 3). The calculation of the VDD charges for both charged systems (Figure 6) shows that the more attractive ▵*V*_elstat_ for *p*‐CF_3_^−^ than for *p*‐OMe^−^ is due to less negatively charged carbon atoms in the former, in line with the electron‐donating character of OMe and the electron‐withdrawing character of CF_3_. In particular, the closest H and C atoms of the aryl ring to the HS^−^ in *p*‐CF_3_^−^ are more positively and less negatively charged, respectively, than in *p*‐OMe^−^, which causes more favorable interaction with the negatively charged HS^−^ group (the distance between the fragments is almost the same in both systems, although slightly shorter for *p*‐CF_3_^−^, so also in line with the better interaction). Finally, S−H stretching frequency shifts of the substituted 2,6‐diarylthiophenols (**1**–**7**) have been tabulated in Table S16. Electron‐donating substituents drive to frequency lowering, whereas electron‐withdrawing ones cause an increase of the frequency.

**Table 3:** Through‐space interaction analyses in simplified models of *para*‐substituted 2,6‐diarylthiophenols and their conjugate bases.^[a]^

	System	Interaction	▵*E*_int_	▵*E*_Pauli_	▵*V*_elstat_	▵*E*_oi_	▵*E*_disp_
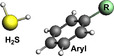	H	H_2_S⋅⋅⋅Aryl(CF_3_)	−0.46	11.35	−5.07	−2.88	−3.87
	p‐OMe	H_2_S⋅⋅⋅Aryl(OMe)	−0.59	11.67	−5.41	−2.98	−3.87
	p‐Me	H_2_S⋅⋅⋅Aryl(Me)	−0.50	11.55	−5.22	−2.94	−3.89
	p‐F	H_2_S⋅⋅⋅Aryl(F)	−0.38	11.34	−5.00	−2.85	−3.86
	p‐Cl	H_2_S⋅⋅⋅Aryl(Cl)	−0.38	11.18	−4.88	−2.81	−3.87
	p‐CF_3_	H_2_S⋅⋅⋅Aryl(CF_3_)	−0.28	10.91	−4.61	−2.69	−3.90
	m‐F	H_2_S⋅⋅⋅Aryl(F)	−0.39	10.88	−4.69	−2.72	−3.86
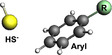	H^−^	HS^−^⋅⋅⋅Aryl(CF_3_)	−2.96	12.44	−4.03	−8.45	−2.93
	p‐OMe^−^	HS^−^⋅⋅⋅Aryl(OMe)	−1.97	12.66	−2.98	−8.71	−2.94
	p‐Me^−^	HS^−^⋅⋅⋅Aryl(Me)	−2.44	12.60	−3.25	−8.84	−2.95
	p‐F^−^	HS^−^⋅⋅⋅Aryl(F)	−5.51	12.55	−6.51	−8.61	−2.93
	p‐Cl^−^	HS^−^⋅⋅⋅Aryl(Cl)	−6.76	12.47	−6.98	−9.31	−2.94
	p‐CF_3_^−^	HS^−^⋅⋅⋅Aryl(CF_3_)	−9.69	12.57	−9.47	−9.82	−2.97
	m‐F^−^	HS^−^⋅⋅⋅Aryl(F)	−5.45	12.31	−6.27	−8.55	−2.93

^[a]^ Values in kcal mol^−1^. Calculated at the ZORA‐BLYP‐D3BJ/TZ2P level of theory. Values correspond to the eclipsed conformation in water.

## 3 Conclusions

In conclusion, our physical‐organic chemistry approach enabled the probing of polar‐π interactions in the series of 2,6‐diarylthiophenols. Hammett analyses, based on measurements of acidity constants and calculations of proton affinity energies, demonstrated that electron‐donating groups at the distant *para* position of the flanking rings in 2,6‐diarylthiophenols lead to weaker acidic character, whereas the presence of electron‐withdrawing groups at the same position results in stronger acids. Our quantum chemical bonding analyses reveal that these trends are the result of predominant intramolecular through‐space polar‐π interactions, whereas the through‐bond effect (resonance and/or inductive) appears to play a minor role in acidity trends. Our bonding analyses of the 2,6‐diarylthiophenols furthermore show that both, S^−^‐π interactions and, to a lesser extent SH‐π interactions, contribute to the overall polar‐π interactions that stabilize 2,6‐diarylthiophenols. This work suggests that the −SH as well as −S^−^ moieties, both representing the existing protonation states of biologically relevant thiols under physiological conditions, can form energetically favorable interactions with electron‐rich aromatic rings that constitute the active/recognition sites in proteins. Thiols may therefore be considered not only as reactive moieties in (bio)molecules, but also as important functional groups that contribute to the stabilization of (bio)molecular structures.

## Experimental Section

### Synthesis of 2,6‐Diarylphenols

To a mixture of 2,6‐dibromophenol acetate and Pd(PPh_3_)_4_ (0.1 equiv) in toluene (4 mL) were added a solution of arylboronic acid (2.5 equiv) in THF (5 mL) and an aqueous solution of Na_2_CO_3_ (2 M, 3 equiv). After it was refluxed at 80 °C under Argon atmosphere for 3 days, the reaction mixture was poured into 20 mL of water and extracted with ethyl acetate (3×30 mL). The combined organic layers were washed with brine, dried over MgSO_4_ and concentrated to give the acetylated 2,6‐diarylphenols. To a solution of acetylated 2,6‐diarylphenols in THF/MeOH/H_2_O was added an aqueous solution of NaOH (5 M, 3 equiv), and the mixture was stirred at room temperature for 15 minutes. The mixture was acidified with 5 % aq. HCl, organic solvent was evaporated, and the aqueous phase extracted with ethyl acetate (3×30 mL). The combined layers were washed with brine (50 mL), dried over Mg_2_SO_4_ and concentrated. The crude product was purified by flash column chromatography on silica gel (PE/EA) to give the 2,6‐diarylphenols **2 a**–**7 a**.

*2,6‐Di(4‐methoxy)phenylphenol (**2** **a**)*. White solid (340 mg, 43 %); ^1^H NMR (400 MHz, CDCl_3_) δ 7.51–7.46 (m, 4H), 7.23 (d, *J*=7.6 Hz, 2H), 7.05–6.98 (m, 5H), 5.37 (s, 1H), 3.86 (s, 6H); ^13^C{^1^H} NMR (101 MHz, CDCl_3_) δ 159.3, 149.6, 130.7, 130.0, 129.7, 128.5, 120.7, 114.5, 55.5.

*2,6‐Di(4‐methyl)phenylphenol (**3 a**)*. White solid (150 mg, 34 %); ^1^H NMR (400 MHz, CDCl_3_) δ 7.51–7.38 (m, 4H), 7.33–7.18 (m, 6H), 7.06–7.00 (m, 1H), 5.40 (s, 1H), 2.41 (s, 6H); ^13^C{^1^H} NMR (101 MHz, CDCl_3_) δ 148.4, 136.4, 133.7, 128.7, 128.5, 128.2, 127.7, 119.6, 20.2.

*2,6‐Di(4‐fluoro)phenylphenol (**4 a**)*. White solid (124 mg, 32 %); ^1^H NMR (400 MHz, CDCl_3_) δ 7.55–7.48 (m, 4H), 7.24 (d, *J*=7.6 Hz, 2H), 7.21–7.12 (m, 4H), 7.05 (t, *J*=7.6 Hz, 1H), 5.23 (s, 1H); ^13^C{^1^H} NMR (101 MHz, CDCl_3_) δ 162.6 (d, *J*=247.3 Hz), 149.4, 133.5(d, *J*=3.4 Hz), 131.2 (d, *J*=8.1 Hz), 130.2, 128.1, 121.0, 116.0 (d, *J*=21.5 Hz); ^19^F NMR (376 MHz, CDCl_3_) δ −114.4.

*2,6‐Di(4‐chloro)phenylphenol (**5 a**)*. White solid (363 mg, 79 %); ^1^H NMR (400 MHz, CDCl_3_) δ 7.54–7.40 (m, 8H), 7.24 (d, *J*=7.6 Hz, 2H), 7.05 (t, *J*=7.6 Hz, 1H), 5.22 (s, 1H); ^13^C{^1^H} NMR (101 MHz, CDCl_3_) δ 149.3, 135.9, 134.0, 130.8, 130.3, 129.2, 127.9, 121.1.

*2,6‐Di(4‐trifluoromethyl)phenylphenol (**6 a**)*. White solid (410 mg, 77 %); ^1^H NMR (400 MHz, CDCl_3_) δ 7.79–7.72 (m, 4H), 7.72–7.65 (m, 4H), 7.32 (d, *J*=7.6 Hz, 2H), 7.13 (t, *J*=7.6 Hz, 1H), 5.23 (s, 1H); ^13^C{^1^H} NMR (101 MHz, CDCl_3_) δ 149.4, 141.2, 130.8, 130.1 (q, *J*=32.6 Hz), 130.0, 128.0, 126.0 (q, *J*=3.8 Hz), 124.3 (q, *J*=272.2 Hz), 121.4; ^19^F NMR (376 MHz, CDCl_3_) δ −62.6.

*2,6‐Di(3‐fluoro)phenylphenol (**7 a**)*. White solid (246 mg, 41 %); ^1^H NMR (400 MHz, CDCl_3_) δ 7.48–7.41 (m, 2H), 7.35–7.31 (m, 2H), 7.30–7.26 (m, 4H), 7.13–7.05 (m, 3H), 5.37 (s, 1H); ^13^C{^1^H} NMR (101 MHz, CDCl_3_) δ 163.0 (d, *J*=246.8 Hz), 149.2, 139.6 (d, *J*=8.2 Hz), 130.4 (d, *J*=8.5 Hz), 130.3, 127.8, 124.9 (d, *J*=2.9 Hz), 121.0, 116.5 (d, *J*=21.8 Hz), 114.7 (d, *J*=21.1 Hz); ^19^F NMR (376 MHz, CDCl_3_) δ −112.3.

### Synthesis of *O*‐(2,6‐Diaryl)phenyl *N,N*‐Dimethylthiocarbamates

To a solution of 2,6‐diarylphenol in anhydrous NMP (0.1 M) at 0 °C under Argon atmosphere was added NaH (2 equiv) in 3 portions every 15 minutes. Then the mixture was stirred for another 30 minutes at 0 °C before dimethylthiocarbamoyl chloride (1.5 equiv) in NMP was added. The mixture was then stirred at 80 °C for 2 hours, quenched with water (15 mL), and extracted with ethyl acetate (3×15 mL). The combined layers were washed with brine, dried over Mg_2_SO_4_ and concentrated. The crude material was purified by flash column chromatography on silica gel (PE/EA) to afford *O*‐(2,6‐diaryl)phenyl *N,N*‐dimethyl thiocarbamates **1 b**–**7 b**.

*O‐(2,6‐diphenyl)phenyl N,N‐dimethylthiolcarbamate (**1 b**)*. White solid (330 mg, 49 %); ^1^H NMR (400 MHz, CDCl_3_) δ 7.53–7.46 (m, 4H), 7.40–7.31 (m, 7H), 7.33–7.25 (m, 2H), 2.99 (s, 3H), 2.94 (s, 3H); ^13^C NMR (101 MHz, CDCl_3_) δ 186.2, 148.1, 138.1, 136.5, 130.1, 129.5, 127.9, 127.3, 126.3, 42.9, 38.3; HRMS (ESI): *m/z* calcd for C_21_H_19_NNaOS [M+Na]^+^: 356.1080, found 356.1093.

*O‐[2,6‐di(4‐methoxylphenyl)phenyl] N,N‐dimethylthiolcarbamate (**2 b**)*. White solid (230 mg, 53 %); ^1^H NMR (400 MHz, CDCl_3_) δ 7.47–7.41 (m, 4H), 7.39–7.30 (m, 3H), 6.96–6.89 (m, 4H), 3.84 (s, 6H), 3.09 (s, 3H), 3.03 (s, 3H); ^13^C{^1^H} NMR (101 MHz, CDCl_3_) δ 186.4, 159.0, 148.3, 136.2, 130.7, 130.6, 129.8, 126.4, 113.5, 55.4, 43.1, 38.4; HRMS (ESI): *m/z* calcd for C_23_H_24_NO_3_S [M+H]^+^: 394.1471, found 394.1453.

*O‐[2,6‐di(4‐methylphenyl)phenyl] N,N‐dimethylthiolcarbamate (**3 b**)*. White solid (110 mg, 56 %); ^1^H NMR (400 MHz, CDCl_3_) δ 7.43–7.33 (m, 7H), 7.23–7.13 (m, 4H), 3.08 (s, 3H), 3.02 (s, 3H), 2.38 (s, 6H); ^13^C{^1^H} NMR (101 MHz, CDCl_3_) 186.5, 148.2, 137.0, 136.5, 135.4, 130.0, 129.4, 128.8, 126.4, 43.0, 38.4, 21.3; HRMS (ESI): *m/z* calcd for C_23_H_23_NNaOS [M+Na]^+^: 384.1393, found 384.1388.

*O‐[2,6‐di(4‐fluorophenyl)phenyl] N,N‐dimethylthiolcarbamate (**4 b**)*. White solid (96 mg, 59 %); ^1^H NMR (400 MHz, CDCl_3_) δ 7.43–7.35 (m, 4H), 7.34–7.25 (m, 3H), 7.03–6.95 (m, 4H), 3.01 (s, 3H), 2.92 (s, 3H); ^13^C{^1^H} NMR (101 MHz, CDCl_3_) δ 186.2, 162.4 (d, *J*=246.4 Hz), 148.2, 135.8, 134.1 (d, *J*=3.4 Hz), 131.2 (d, *J*=8.0 Hz), 130.3, 126.5, 115.0 (d, *J*=21.4 Hz), 43.1, 38.4; ^19^F NMR (376 MHz, CDCl_3_) δ −115.1; HRMS (ESI): *m/z* calcd for C_21_H_18_F_2_NOS [M+H]^+^: 370.1072, found 370.1067.

*O‐[2,6‐di(4‐chlorophenyl)phenyl] N,N‐dimethylthiolcarbamate (**5 b**)*. White solid (216 mg, 47 %); ^1^H NMR (400 MHz, CDCl_3_) δ 7.44–7.32 (m, 11H), 3.09 (s, 3H), 3.01 (s, 3H); ^13^C{^1^H} NMR (101 MHz, CDCl_3_) δ 186.02, 148.0, 136.5, 135.6, 133.6, 130.7, 130.4, 128.3, 126.6, 43.2, 38.4; HRMS (ESI): *m/z* calcd for C_21_H_18_Cl_2_NOS [M+H]^+^: 402.0481, found 402.0467.

*O‐[2,6‐di(4‐trifluoromethylphenyl)phenyl] N,N‐dimethylthiolcarbamate (**6 b**)*. White solid (170 mg, 36 %); ^1^H NMR (400 MHz, CDCl_3_) δ 7.69–7.58 (m, 8H), 7.49–7.37 (m, 3H), 3.05 (s, 3H), 2.99 (s, 3H); ^13^C{^1^H} NMR (101 MHz, CDCl_3_) δ 185.9, 148.0, 141.6, 135.6, 130.8, 129.9, 129.4 (q, *J*=32.4 Hz), 126.8, 125.1 (q, *J*=3.7 Hz), 124.4 (q, 272.1 Hz), 43.2, 38.4; ^19^F NMR (376 MHz, CDCl_3_) δ −62.4; HRMS (ESI): *m/z* calcd for C_23_H_18_F_6_NOS [M+H]^+^: 470.1008, found 470.1024.

*O‐[2,6‐di(3‐fluorophenyl)phenyl] N,N‐dimethylthiolcarbamate (**7 b**)*. White solid (180 mg, 56 %); ^1^H NMR (400 MHz, CDCl_3_) δ 7.42–7.38 (m, 3H), 7.37–7.31 (m, 2H), 7.27 (dt, *J*=7.7, 1.3 Hz, 2H), 7.23 (ddd, *J*=10.0, 2.6, 1.5 Hz, 2H), 7.03 (tdd, *J*=8.3, 2.6, 1.1 Hz, 2H), 3.09 (s, 3H), 3.04 (s, 3H); ^13^C{^1^H} NMR (101 MHz, CDCl_3_) δ 186.0, 162.5 (d, *J*=245.3 Hz), 148.0, 140.1 (d, *J*=8.1 Hz), 130.5, 129.6 (d, *J*=8.5 Hz), 126.6, 125.4 (d, *J*=2.9 Hz), 116.5 (d, *J*=22.3 Hz), 114.4 (d, *J*=21.0 Hz), 43.2, 38.4; ^19^F NMR (376 MHz, CDCl_3_) δ −113.7; HRMS (ESI): *m/z* calcd for C_21_H_18_F_2_NOS [M+H]^+^: 370.1072, found 370.1054.

### Synthesis of *S*‐(2,6‐Diaryl)phenyl *N,N*‐Dimethylthiolcarbamates

The *O*‐(2,6‐diaryl)phenyl *N,N*‐dimethylthiocarbamates were dissolved in NMP (0.1 M) and heated at 300 °C under microwave for 3 hours. After cooling to room temperature, the solvent was evaporated to give a dark brown oil. The crude product was purified by flash column chromatography on silica gel (PE/EA) to afford *S*‐(2,6‐diaryl)phenyl *N,N*‐dimethylthiolcarbamates **1 c**–**7 c**.

*S‐(2,6‐diphenyl)phenyl N,N‐dimethylthiolcarbamate (**1 c**)*. Colorless oil (250 mg, 76 %); ^1^H NMR (400 MHz, CDCl_3_) δ 7.50–7.26 (m, 13H), 2.65 (brs, 6H); ^13^C{^1^H} NMR (101 MHz, CDCl_3_) δ 166.8, 148.7, 142.2, 129.8, 129.6, 129.3, 127.5, 127.0, 126.7, 37.0; HRMS (ESI): *m/z* calcd for C_21_H_19_NNaOS [M+Na]^+^: 356.1080, found 356.1074.

*S‐[2,6‐di(4‐methoxylphenyl)phenyl] N,N‐dimethylthiolcarbamate (**2 c**)*. White solid (182 mg, 79 %); ^1^H NMR (400 MHz, CDCl_3_) δ 7.47–7.42 (dd, *J*=8.2, 6.9 Hz, 1H), 7.36–7.31 (m, 6H), 6.94–6.87 (m, 4H), 3.85 (s, 6H), 2.73 (brs, 6H); ^13^C{^1^H} NMR (101 MHz, CDCl_3_) δ 167.0, 158.7, 148.3, 134.8, 130.7, 129.8, 129.2, 127.0, 112.9, 55.3, 36.9; HRMS (ESI): *m/z* calcd for C_23_H_23_NNaO_3_S [M+Na]^+^: 416.1291, found 416.1300.

*S‐[2,6‐di(4‐methylphenyl)phenyl] N,N‐dimethylthiolcarbamate (**3 c**)*. Colorless oil (70 mg, 64 %); ^1^H NMR (400 MHz, CDCl_3_) δ 7.49–7.43 (m, 1H), 7.37–7.28 (m, 6H), 7.22–7.16 (m, 4H), 2.71 (brs, 6H), 2.40 (s, 6H); ^13^C{^1^H} NMR (101 MHz, CDCl_3_) δ 167.2, 148.8, 139.5, 136.7, 129.9, 129.6, 129.3, 128.3, 126.9, 37.1, 21.4; HRMS (ESI): *m/z* calcd for C_23_H_24_NOS [M+H]^+^: 362.1573, found 362.1573.

*S‐[2,6‐di(4‐fluorophenyl)phenyl] N,N‐dimethylthiolcarbamate (**4 c**)*. Colorless oil (28 mg, 63 %); ^1^H NMR (400 MHz, CDCl_3_) δ 7.48 (m, 1H), 7.39–7.32 (m, 6H), 7.10–7.02 (m, 4H), 2.73 (brs, 6H); ^13^C{^1^H} NMR (101 MHz, CDCl_3_) δ 166.6, 162.3 (d, *J*=245.9 Hz), 147.9, 138.1 (d, *J*=3.4 Hz), 131.3 (d, *J*=8.0 Hz), 130.1, 129.5, 127.0, 114.5 (d, *J*=21.4 Hz), 37.1; HRMS (ESI): *m/z* calcd for C_21_H_17_F_2_NNaOS [M+Na]^+^: 392.0891, found 392.0891.

*S‐[2,6‐di(4‐chlorophenyl)phenyl] N,N‐dimethylthiolcarbamate (**5 c**)*. Colorless oil (170 mg, 47 %); ^1^H NMR (400 MHz, CDCl_3_) δ 7.52–7.46 (m, 1H), 7.38–7.30 (m, 10H), 2.74 (brs, 6H); ^13^C{^1^H} NMR (101 MHz, CDCl_3_) δ 166.5, 147.8, 140.5, 133.3, 131.0, 130.1, 129.7, 127.8, 126.6, 37.1; HRMS (ESI): *m/z* calcd for C_21_H_17_Cl_2_NNaOS [M+Na]^+^: 424.0300, found 424.0301.

*S‐[2,6‐di(4‐trifluoromethylphenyl] N,N‐dimethylthiolcarbamate (**6 c**)*. Colorless oil (80 mg, 47 %); ^1^H NMR (400 MHz, CDCl_3_) δ 7.68–7.61 (m, 4H), 7.59–7.48 (m, 5H), 7.42–7.37 (m, 2H), 2.73 (s, 3H), 2.69 (s, 3H); ^13^C{^1^H} NMR (101 MHz, CDCl_3_) δ 166.1, 147.7, 145.5, 130.3, 130.1, 129.8, 129.6 (q, *J*=32.1 Hz) 124.6 (q, *J*=3.8 Hz), 124.5 (q, *J*=272.1 Hz), 37.0; ^19^F NMR (376 MHz, CDCl_3_) δ −62.4; HRMS (ESI): *m/z* calcd for C_23_H_17_F_6_NNaOS [M+Na]^+^: 492.0827, found 492.0855.

*S‐[2,6‐di(3‐fluorophenyl)phenyl] N,N‐dimethylthiolcarbamate (**7 c**)*. White foam (49 mg, 27 %); ^1^H NMR (400 MHz, CDCl_3_) δ 7.51 (dd, *J*=8.2, 7.0 Hz, 1H), 7.41–7.30 (m, 4H), 7.21–7.11 (m, 4H), 7.05 (tdd, *J*=8.6, 2.6, 1.0 Hz, 2H), 2.75 (s, 6H); ^13^C{^1^H} NMR (101 MHz, CDCl_3_) δ 166.5, 162.2 (d, *J*=245.4 Hz), 147.7 (d, *J*=1.9 Hz), 144.1 (d, *J*=8.1 Hz), 130.1, 129.6, 129.1 (d, *J*=8.3 Hz), 126.6, 125.5 (d, *J*=2.9 Hz), 116.7 (d, *J*=22.0 Hz), 114.1 (d, *J*=21.0 Hz), 37.0; ^19^F NMR (376 MHz, CDCl_3_) δ −114.1; HRMS (ESI): *m/z* calcd for C_21_H_17_F_2_NNaOS [M+Na]^+^: 392.0891, found 392.0891.

### Synthesis of 2,6‐Diarylthiophenols

To a flask filled of LiAlH_4_ (10 equiv), a solution of *S*‐(2,6‐diaryl)phenyl *N,N*‐dimethylthiolcarbamates in anhydrous THF (0.1 M) was added dropwise at 0 °C under Argon atmosphere. Then the mixture was heated under reflux for 2 hours. The reaction was cooled to 0 °C, and methanol (1 mL) was added slowly to quench the reaction. Then H_2_SO_4_ (6 M, 3 mL) was added and the mixture was stirred at room temperature for 30 minutes. The mixture was filtered, and filtrate was extracted with ethyl acetate. The combined layers were washed with brine, dried over Mg_2_SO_4_ and concentrated. The crude product was purified by flash column chromatography on silica gel (PE/DCM) to yield 2,6‐diaryl(thiophenol)s **1**–**7**.

*2,6‐diphenyl(thiophenol) (**1**)*. White solid (64 mg, 81 %); mp 67–68 °C; ^1^H NMR (400 MHz, CDCl_3_) δ 7.44 (m, 8H), 7.41–7.35 (m, 2H), 7.21–7.18 (m, 3H), 3.41 (s, 1H); ^13^C{^1^H} NMR (101 MHz, CDCl_3_) δ 141.5, 140.9, 130.8, 129.4, 129.4, 128.6, 127.8, 124.5; HRMS (GC‐TOF) [M^+^] m/z calcd for C_18_H_14_S 262.0816; found, 262.1639.

*2,6‐di(4‐methoxyphenyl)benzenethiol (**2**)*. White solid (53 mg, 73 %); mp 98–99 °C; ^1^H NMR (400 MHz, CDCl_3_) δ 7.42–7.34 (m, 4H), 7.19–7.16 (m, 3H), 7.04–6.94 (m, 4H), 3.86 (s, 6H), 3.47 (s, 1H); ^13^C{^1^H} NMR (101 MHz, CDCl_3_) δ 159.3, 140.6, 134.1, 130.7, 129.5, 124.5, 114.1, 55.5; HRMS (GC‐TOF) [M^+^] m/z calcd for C_20_H_18_O_2_S 322.1028; found, 322.2073.

*2,6‐di(4‐methylphenyl)benzenethiol (**3**)*. White solid (25 mg, 78 %); mp 108–109°C; ^1^H NMR (400 MHz, CDCl_3_) δ 7.37–7.30 (m, 4H), 7.31–7.23 (m, 4H), 7.22–7.15 (m, 3H), 3.46 (s, 1H), 2.41 (s, 6H); ^13^C{^1^H} NMR (101 MHz, CDCl_3_) δ 141.0, 138.8, 137.6, 131.1, 129.4, 129.4, 124.6, 21.4; HRMS (GC‐TOF) [M^+^] m/z calcd for C_20_H_18_S 290.1129; found, 290.2091.

*2,6‐di(4‐fluorophenyl)benzenethiol (**4**)*. White solid (16 mg, 70 %); mp 73–74 °C; ^1^H NMR (400 MHz, CDCl_3_) δ 7.45–7.37 (m, 4H), 7.24–7.11 (m, 7H), 3.33 (s, 1H); ^13^C{^1^H} NMR (101 MHz, CDCl_3_) δ 162.6 (d, *J*=247.2 Hz), 140.1, 137.4 (d, *J*=3.4 Hz), 131.2 (d, *J*=8.1 Hz), 129.8, 124.8, 115.7 (d, *J*=21.5 Hz); ^19^F NMR (376 MHz, CDCl_3_) δ −114.2; HRMS (GC‐TOF) [M+] m/z calcd for C_18_H_12_F_2_S 298.0628; found, 298.1617.

*2,6‐di(4‐chlorophenyl)benzenethiol (**5**)*. White solid (13 mg, 64 %); mp 165–168 °C; ^1^H NMR (400 MHz, CDCl_3_) δ 7.46–7.41 (m, 4H), 7.40–7.35 (m, 4H), 7.25–7.16 (m, 3H), 3.33 (s, 1H); ^13^C{^1^H} NMR (101 MHz, CDCl_3_) δ 140.0, 139.8, 134.1, 130.9, 130.7, 129.8, 129.0, 124.9; HRMS (GC‐TOF) [M+] m/z calcd for C_18_H_12_Cl_2_S 330.0037; found, 330.1085.

*2,6‐di(4‐trifluoromethylphenyl)benzenethiol (**6**)*. White solid (45 mg, 66 %); mp 143–145 °C; ^1^H NMR (400 MHz, CDCl_3_) δ 7.78–7.69 (m, 4H), 7.62–7.54 (m, 4H), 7.30–7.25 (m, 1H), 7.24–7.20 (m, 2H), 3.24 (s, 1H); ^13^C{^1^H} NMR (101 MHz, CDCl_3_) δ 144.8, 140.0, 130.3 (q, *J*=32.5 Hz), 130.3, 130.0, 130.0, 125.8 (q, *J*=3.7 Hz), 125.2, 124.3 (q, *J*=272.3 Hz); ^19^F NMR (376 MHz, CDCl_3_) δ −62.6; HRMS (GC‐TOF) [M+] m/z calcd for C_20_H_12_F_6_S 398.0564; found, 398.2094.

2*,6‐di(3‐fluorophenyl)benzenethiol (**7**)*. White solid (24 mg, 60 %); mp 99–100 °C; ^1^H NMR (400 MHz, CDCl_3_) δ 7.43 (td, *J*=8.0, 5.9 Hz, 2H), 7.25–7.19 (m, 5H), 7.17 (dd, *J*=2.6, 1.6 Hz, 1H), 7.14 (dd, *J*=2.6, 1.6 Hz, 1H), 7.13–7.07 (m, 2H), 3.37 (s, 1H); ^13^C{^1^H} NMR (101 MHz, CDCl_3_) δ 162.9 (d, *J*=247.1 Hz), 143.5 (d, *J*=7.7 Hz), 139.9 (d, *J*=2.0 Hz), 130.6, 130.3 (d, *J*=8.4 Hz), 129.8, 125.3 (d, *J*=3.0 Hz), 124.9, 116.6 (d, *J*=21.7 Hz), 114.9 (d, *J*=21.1 Hz); ^19^F NMR (376 MHz, CDCl_3_) δ −112.5; HRMS (GC‐TOF) [M+] m/z calcd for C_18_H_12_F_2_S 298.0628; found, 298.1594.

### Quantum Chemical Analyses

All calculations were carried out with the Amsterdam Density Functional (ADF) program using dispersion‐corrected density functional theory at the BLYP‐D3BJ/TZ2P level of theory.^14^, ^15^ The effect of solvation in water was simulated by means of the Conductor like Screening Model (COSMO) of solvation as implemented in ADF. The approach has been benchmarked against highly correlated post‐Hartree‐Fock methods and experimental data and was found to work reliably.^12^, ^16^, ^17^ The bonding mechanism of hydrogen sulfide (taken from thiophenol) or bisulfide anion (taken from unprotonated thiophenol) with the two substituted benzene rings (taken from aryl rings) was analyzed within the framework of quantitative Kohn‐Sham molecular orbital theory^18^ in combination with a quantitative energy decomposition analysis (EDA)^18^ in the gas phase. The interaction energy ▵*E*_int_ between these fragments is decomposed into the classical electrostatic attraction ▵*V*_elstat_, Pauli repulsion ▵*E*_Pauli_ between occupied orbitals, stabilizing orbital interactions ▵*E*_oi_, and dispersion ▵*E*_disp._ Atomic charges were computed with the Voronoi deformation density (VDD) method.^19^

### Energy Decomposition Analysis Methodology

To analyze the through‐space interactions between the bisulfide group and the *para*‐substituted aryl rings in the 2,6‐diarylthiophenols, the central benzene ring was removed, and also the aryl moiety to which the H of the SH group is not pointing to. Thus, the remaining two moieties (the bisulfide radical and the *para*‐substituted phenyl radical to which the SH is pointing to) were kept frozen to their geometry and position they had in the complete thiophenol system. The two radical positions were terminated with hydrogen atoms. It is worth noting that only the bond lengths of these three hydrogen atoms were geometrically optimized (angles were also constrained) at the ZORA‐BLYP‐D3BJ/TZ2P level; other atoms were kept frozen to keep the structure of 2,6‐diarylthiophenol system. Next, the added proton to the bisulfide radical was rotated through the other S−H bond to be as far as possible from the aryl ring, thus avoiding spurious S−H⋅⋅⋅H−C steric repulsion that is not present in the original compound.

### X‐ray Crystallography

The data for compound **4** were collected at 100(1)K on a Synergy, Dualflex, AtlasS2 diffractometer using Cu*K*α radiation (λ=1.54184 Å) and the *CrysAlis PRO* 1.171.40.29a suite. Using SHELXLE and Olex2 the structure was solved by dual space methods (SHELXT) and refined on *F* ^2^ using all the reflections (SHELXL‐2018/3). All the non‐hydrogen atoms were refined using anisotropic atomic displacement parameters and hydrogen atoms bonded to carbon inserted at calculated positions using a riding model. For compound **6**, reflections were measured on a Bruker D8 Quest diffractometer with sealed tube and Triumph monochromator (λ=0.71073 Å). Software package used for the intensity integration was Saint. Absorption correction was performed with SADABS. The structure was solved with direct methods using SHELXT. Least‐squares refinement was performed with SHELXL‐2014 against |F_h o| 2 of all reflections. Non‐hydrogen atoms were refined freely with anisotropic displacement parameters. Hydrogen atoms were placed on calculated positions or located in difference Fourier maps. All calculated hydrogen atoms were refined with a riding model. Data, data collection and structure refinement details are summarised in Tables S1 and S5. CCDCs 1980288 and 1980799 contain the supplementary crystallographic data for this paper. These data can be obtained free of charge from The Cambridge Crystallographic Data Centre via www.ccdc.cam.ac.uk/data_request/cif.

## Supporting information

NMR spectra, p*K*_a_ measurements, IR data, X‐ray crystallography data, computational studies. Crystallographic data for **4** and **6**.

## Conflict of interest

The authors declare no conflict of interest.

## Supporting information

As a service to our authors and readers, this journal provides supporting information supplied by the authors. Such materials are peer reviewed and may be re‐organized for online delivery, but are not copy‐edited or typeset. Technical support issues arising from supporting information (other than missing files) should be addressed to the authors.

Supplementary

Supplementary

Supplementary
