# Supplementary material for: Through‐Space Polar‐π Interactions in 2,6‐Diarylthiophenols
Source: Chemphyschem. 2020 May 8;21(11):1092–100. doi: 10.1002/cphc.202000132 (PMC7318691; doi:10.1002/cphc.202000132)
Supplement: Supplementary file 3 — Supplementary [file CPHC-21-1092-s003.pdf]

ChemPhysChem

Supporting Information

Through-Space Polar-π Interactions in 2,6-Diarylthiophenols

Jie Jian[+], Jordi Poater[+], Roel Hammink[+], Paul Tinnemans, Christine J. McKenzie, F. Matthias Bickelhaupt,* and Jasmin Mecinović*© 2020 The Authors. Published by Wiley-VCH Verlag GmbH & Co. KGaA. This is an open access article under the terms of the Creative Commons Attribution License, which permits use, distribution and reproduction in any medium, provided the original work is properly cited.An invited contribution to a Special Collection on Molecular Recognition

TABLE S1

Convergence criteria and other computational details on the DFT electronic structure calculations based on projector-augmented wave (PAW) or Gaussian type orbital (GTO) basis sets.

Job type	Parameter	Basis set type PAW	Basis set type GTO
Single-point energy	SCF cycle convergence	10^{-8} eV	$2.7{\cdot}10^{-5}$ eV
	k-points mesh points	$20/a_i$ to $30/a_i$	8
Unit-cell optimization	SCF cycle convergence	10^{-8} eV	$2.7{\cdot}10^{-7}$ eV
	geometry optimization	10^{-6} eV	$2.7{\cdot}10^{-6}$ eV
Phonons	SCF cycle convergence	10^{-8} eV	-
	geometry optimization	10^{-6} eV·Å^{-1}	-
	SCF cycle convergence for perturbed supercells	10^{-8} eV	-
	k-points mesh points	2	-

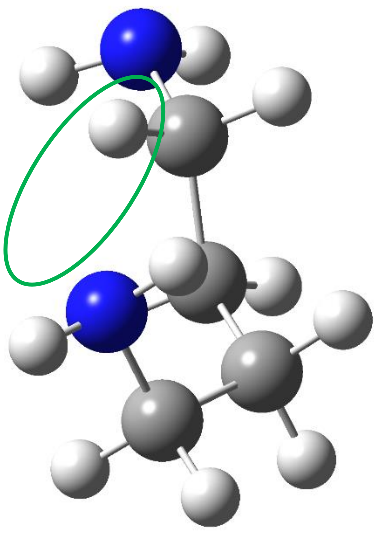

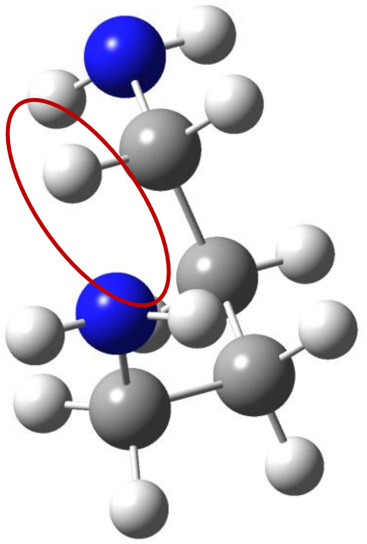

gg’dtt conformer: stable

gd’gtt conformer: unstable

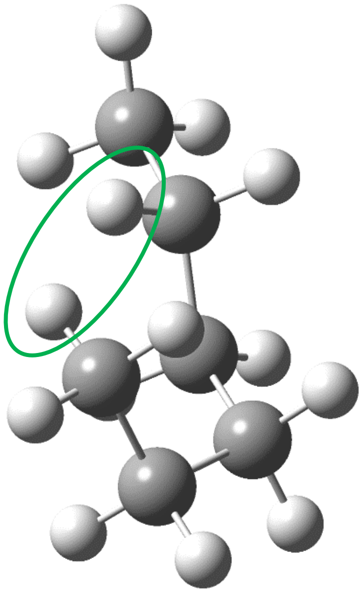

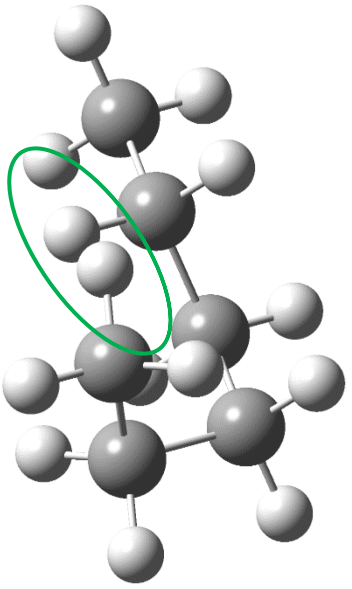

g’dt conformer: stable

d’gt conformer: stable

FIGURE S1. Additional stability rule for amino-terminated alkyl chain. While alkanes show pentane effect (existence of gd’ and dg’ conformers) with any orientation of the methyl group, the terminal amino group does not enable existence of two distinct conformers if the lone pair is oriented against the 4-hydrogen.

TABLE S2.

Atomic multipoles parametrized for C_n-diamines. All multipoles are compatible with the standard setting of the Amoeba force field. Charge unit is the elementary charge, distance unit is Å. First row lists the definition of the coordinates of the local atomic multipole frame, defined through the common Amoeba conventions, and the partial atomic charge, second row lists the atomic dipole with respect to the local frame, last three rows contain the atomic quadrupole.

S1.1 C_2-diamine

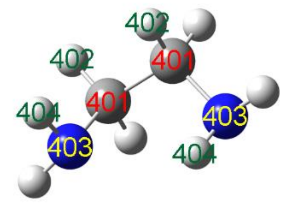

multipole	401	403	401	0.05718		
				0.02975	0.00000	0.56402
				-1.23925		
				0.00000	-0.44719	
				0.58312	0.00000	1.68644
multipole	402	401	403	0.15576		
				0.22986	0.00000	-0.11167
				0.14122		
				0.00000	0.08772	
				0.21718	0.00000	-0.22894
multipole	403	401	404	-0.16039		
				0.00634	0.00000	0.56629
				-0.31015		
				0.00000	-0.53677	
				-0.01405	0.00000	0.84692
multipole	404	403	401	-0.10415		
				-0.26291	0.00000	-0.83581
				0.39824		
				0.00000	0.42373	
				-0.44201	0.00000	-0.82197

S1.2 C_3-diamine

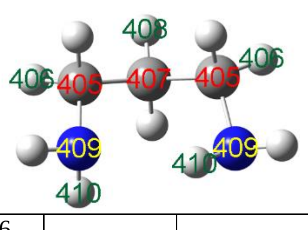

multipole	405	409	407	-0.20656		
				-0.33550	0.00000	0.33279
				-1.05357		
				0.00000	0.59112	
				0.53684	0.00000	0.46245
multipole	406	405	409	0.10742		
				0.09505	0.00000	-0.30929
				0.31084		
				0.00000	0.16649	
				0.08595	0.00000	-0.47733
multipole	407	-405	-405	-0.01190		
				0.00000	0.00000	-0.80078
				-0.56680		
				0.00000	2.12335	
				0.00000	0.00000	-1.55655

S1.3 C_4-diamine

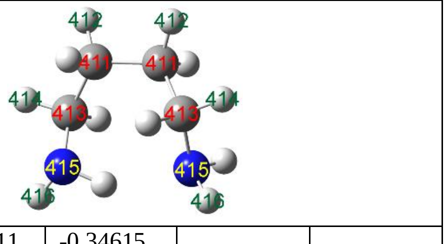

multipole	411	413	411	-0.34615		
				-0.65893	0.00000	-0.04352
				-0.96396		
				0.00000	1.48856	
				0.43928	0.00000	-0.52460
multipole	412	411	411	0.14285		
				0.01327	0.00000	-0.28469
				0.39814		
				0.00000	-0.02666	
				-0.14456	0.00000	-0.37148
multipole	413	415	411	-0.09333		
				-0.36419	0.00000	0.70508
				-1.63082		
				0.00000	0.44835	
				0.59473	0.00000	1.18247

multipole	408	407	405	0.06666			multipole	414	413	415	0.18302		
				0.00516	0.00000	-0.30359					0.00453	0.00000	-0.10977
				0.21067							0.10198		
				0.00000	0.20786						0.00000	0.10067	
				0.03102	0.00000	-0.41853					-0.14206	0.00000	-0.20265
multipole	409	405	410	-0.38319			multipole	415	413	416	-0.48763		
				-0.03732	0.00000	0.90281					-0.00025	0.00000	0.72454
				-0.10409							0.28835		
				0.00000	-0.12869						0.00000	0.14396	
				-0.17176	0.00000	0.23278					-0.03474	0.00000	-0.43231
multipole	410	409	405	0.15710			multipole	416	415	413	0.13768		
				-0.36054	0.00000	-0.41306					-0.35933	0.00000	-0.43844
				0.18934							0.39846		
				0.00000	0.10435						0.00000	-0.00730	
				-0.60399	0.00000	-0.29369					-0.57482	0.00000	-0.39116

**S1.4 C$_5$-diamine**				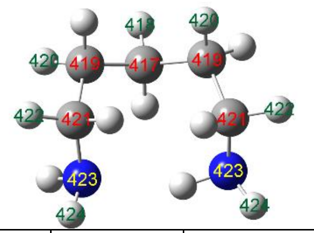			**S1.5 C$_6$-diamine**				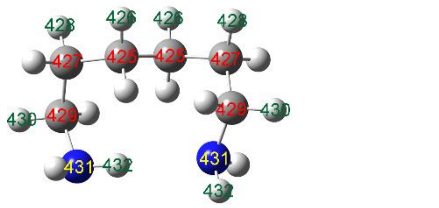		
multipole	417	-419	-419	-0.06214			multipole	425	-427	-425	-0.23225		
				0.00000	0.00000	-0.60836					-0.33109	0.00000	-0.47801
				-1.45333							-0.67911		
				0.00000	1.17223						0.00000	1.65910	
				0.00000	0.00000	0.28110					0.58023	0.00000	-0.97999
multipole	418	417	419	-0.01812			multipole	426	425	427	0.16756		
				0.00290	0.00000	-0.46998					-0.20822	0.00000	-0.40084
				0.25517							0.29433		
				0.00000	0.05102						0.00000	0.08602	
				-0.00728	0.00000	-0.30619					-0.36010	0.00000	-0.38035
multipole	419	421	417	-0.45421			multipole	427	429	425	-0.48021		
				-0.86833	0.00000	0.13061					-0.80017	0.00000	0.27686
				-1.35321							-1.94385		
				0.00000	1.66485						0.00000	1.55149	
				0.54957	0.00000	-0.31164					0.16413	0.00000	0.39236
multipole	420	419	417	0.18048			multipole	428	427	425	0.20871		
				0.13000	0.00000	-0.03493					0.04302	0.00000	-0.34806
				0.31601							0.53881		
				0.00000	-0.13338						0.00000	0.13944	
				0.11609	0.00000	-0.18263					-0.06958	0.00000	-0.67825
multipole	421	423	419	0.17953			multipole	429	431	427	0.05335		
				0.00691	0.00000	1.04492					-0.39025	0.00000	0.91617
				-1.67062							-1.37827		
				0.00000	-0.81503						0.00000	0.27567	
				0.42496	0.00000	2.48565					0.60670	0.00000	1.10260
multipole	422	421	423	0.18062			multipole	430	429	431	0.13189		
				0.14066	0.00000	-0.12655					0.04671	0.00000	-0.19148
				-0.01714							0.22163		
				0.00000	0.15322						0.00000	0.08398	
				0.11138	0.00000	-0.13608					0.00289	0.00000	-0.30561
multipole	423	421	424	-0.48357			multipole	431	429	432	-0.64569		
				-0.01583	0.00000	0.59055					0.01324	0.00000	0.61679
				-0.38054							0.37544		
				0.00000	-0.44656						0.00000	0.04857	
				-0.04739	0.00000	0.82710					0.05224	0.00000	-0.42401
multipole	424	423	421	0.04262			multipole	432	431	429	0.14425		
				-0.24595	0.00000	-0.58969					-0.36297	0.00000	-0.43080
				0.31967							0.34514		
				0.00000	0.21821						0.00000	0.06853	
				-0.36329	0.00000	-0.53788					-0.50476	0.00000	-0.41367

S1.6 C_7-diamine

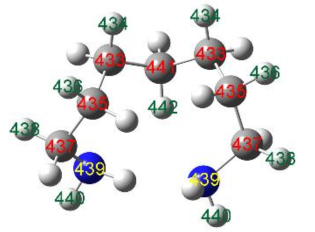

multipole	433	-435	-441	-0.13557		
				-0.53271	0.00000	-0.58359
				-0.42421		
				0.00000	2.13110	
				0.37996	0.00000	-1.70689
multipole	434	433	435	0.21673		
				-0.08037	0.00000	-0.41753
				0.56847		
				0.00000	0.02922	
				0.07201	0.00000	-0.59769
multipole	435	437	433	-0.62404		
				-0.86189	0.00000	0.52073
				-1.58196		
				0.00000	1.27064	
				1.03308	0.00000	0.31132
multipole	436	435	433	0.15288		
				0.07153	0.00000	-0.27467
				0.43921		
				0.00000	0.10124	
				0.00839	0.00000	-0.54045
multipole	437	439	435	0.05339		
				-0.40762	0.00000	0.86692
				-0.53893		
				0.00000	0.99252	
				0.55525	0.00000	-0.45359
multipole	438	437	439	0.07719		
				-0.08103	0.00000	-0.13768
				0.28420		
				0.00000	-0.05644	
				-0.07381	0.00000	-0.22776
multipole	439	437	440	-0.53834		
				0.00078	0.00000	0.57782
				0.15705		
				0.00000	-0.02640	
				0.04680	0.00000	-0.13065
multipole	440	439	437	0.14562		
				-0.29493	0.00000	-0.38381
				0.28787		
				0.00000	-0.00466	
				-0.43331	0.00000	-0.28321
multipole	441	-433	-433	-0.12010		
				0.00000	0.00000	-0.73420
				-0.23482		
				0.00000	1.34568	
				0.00000	0.00000	-1.11086
multipole	442	441	433	0.11977		
				0.00469	0.00000	-0.50693
				0.17003		
				0.00000	0.36150	
				-0.04186	0.00000	-0.53153

S1.7 C_8-diamine

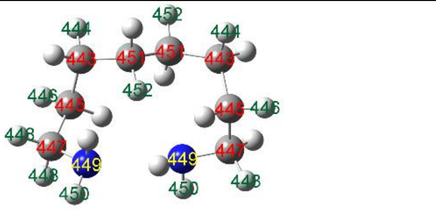

multipole	443	-445	-451	-0.45609		
				-0.12902	0.00000	-0.58300
				-0.37130		
				0.00000	1.43321	
				0.00462	0.00000	-1.06191
multipole	444	443	445	0.18601		
				0.04152	0.00000	-0.27722
				0.38299		
				0.00000	0.05996	
				0.11701	0.00000	-0.44295
multipole	445	447	443	-0.38109		
				-0.54574	0.00000	0.00075
				-0.92501		
				0.00000	1.35376	
				0.02304	0.00000	-0.42875
multipole	446	445	443	0.17238		
				0.00279	0.00000	-0.26173
				0.27657		
				0.00000	0.14122	
				-0.08754	0.00000	-0.41779
multipole	447	449	445	0.01688		
				-0.45668	0.00000	0.73132
				-0.80407		
				0.00000	1.35426	
				0.60026	0.00000	-0.55019
multipole	448	447	449	0.09606		
				-0.04472	0.00000	-0.19818
				0.34170		
				0.00000	0.02356	
				-0.06359	0.00000	-0.36526
multipole	449	447	450	-0.55413		
				0.00710	0.00000	0.66373
				0.42438		
				0.00000	0.37769	
				0.05237	0.00000	-0.80207
multipole	450	449	447	0.13170		
				-0.45187	0.00000	-0.45038
				0.31588		
				0.00000	0.04882	
				-0.72188	0.00000	-0.36470
multipole	451	-443	-451	-0.14161		
				-0.18844	0.00000	-0.28966
				-0.74163		
				0.00000	1.35090	
				0.04484	0.00000	-0.60927
multipole	452	451	443	0.17187		
				-0.07727	0.00000	-0.31083
				0.16569		
				0.00000	0.16480	
				-0.07648	0.00000	-0.33049

TABLE S3

Comparison of the numbers of dynamically stable conformers estimated by empirical rules with the numbers refined by the quantum-chemical geometry optimizations and frequency calculations.

C_n-diamine	Upper bound[a]	Best guess[b]	B3LYP-D3[c]	DFTBA[c]	GFN1-xTB	GFN2-xTB
C_2	10	10	9	10	10	10
C_3	28	25	25	27	25	25
C_4	88	79	79	80	74	88
C_5	265	229	227	237	223	227
C_6	856	734	733	768	712	703
C_7	2710	2289	-	2417	2656	2683
C_8	8767	7291	-	7693	8567	8531

[a] Upper bound for the number of stable conformers based on the strong enumeration rules. These conformers were optimized by the B3LYP-D3 functional and DBTBA method.

[b] Estimation of real number of stable conformers based on strong and very weak enumeration rules that were demonstrated to predict well the number of conformers stable at the B3LYP-D3 functional for n-alkanes.

[c] Conformers successfully optimized at the B3LYP-D3/6-311+G(2df,p) and DBTBA levels of theory, respectively.

TABLE S4

Performance of various fast lower-tier approaches for predictions of the relative electronic energies of individual conformers of isolated C_n-diamine molecules. Root-mean-square errors (RMSE, kJ mol^{-1}) of the conformation energies are calculated with respect to the CCSD(T)/CBS electronic energies obtained for molecular geometries optimized at the B3LYP-D3/6-311+G(2df,p) level of theory.

C_n-diamine	RMSE						
	Amoeba-L[a]	Amoeba-LC[b]	Amoeba-LCV[c]	DFTBA	GFN1-xTB	GFN2-xTB	B3LYP-D3
C_2	3.6	4.3	2.9	3.2	4.4	5.2	0.7
C_3	39	19	8.2	3.4	5.2	4.5	0.7
C_4	38	21	13	2.5	5.9	7.1	0.7
C_5	16	18	12	2.1	5.6	6.3	0.7
C_6	20	27	16	3.1	5.6	4.5	1.9

[a] Atomic multipoles parametrized with respect to the all-trans conformers, constraining the monopoles during the second refinement.

[b] Atomic multipoles parametrized with respect to both the all-trans and the most stable cyclic conformers, constraining the monopoles of the all-trans one during the second refinement.

[c] Atomic multipoles parametrized with respect to both the all-trans and the most stable cyclic conformers, varying the monopoles during the second refinement.

TABLE S5

Unit-cell parameters optimized with respect to the static electronic energy. All calculations were performed with the D3(BJ) dispersion correction. ATM three-body dispersion was included except for PAW calculations.

C_2-diamine					
	a, Å	*b*, Å	*c*, Å	*β*	*V*, Å^3
Experiment	5.047	7.155	5.475	115.35	178.66
Pseudo-experiment	-	-	-	-	166.3
vdW-DF2/PAW	5.097	7.234	5.467	113.98	184.2
PBE/PAW	5.268	7.522	5.440	113.48	197.7
PBE/pob-TZVP	4.955	6.918	5.303	114.04	166.0
PBE/pob-TZVP-rev2	4.944	6.952	5.296	114.03	166.2
B3LYP/pob-TZVP	4.918	6.872	5.311	114.40	163.5
B3LYP/pob-TZVP-rev2	4.906	6.891	5.310	114.32	163.6

C_3-diamine				
	a, Å	*b*, Å	*c*, Å	*V*, Å^3
Experiment	12.619	6.130	5.907	456.9
Pseudo-experiment	-	-	-	425.3
vdW-DF2/PAW	12.871	6.059	5.952	464.2
PBE/PAW	12.644	6.256	5.977	472.8
PBE/pob-TZVP	12.515	5.914	5.683	420.6
PBE/pob-TZVP-rev2	12.521	5.929	5.701	423.2
B3LYP/pob-TZVP	12.545	5.838	5.683	416.2
B3LYP/pob-TZVP-rev2	12.542	5.855	5.705	419.0
C_4-diamine				
	a, Å	*b*, Å	*c*, Å	*V*, Å^3
Experiment	6.552	5.738	14.528	546.2
Pseudo-experiment	-	-	-	508.4
vdW-DF2/PAW	6.304	5.928	15.112	564.7
PBE/PAW	7.362	5.640	15.056	625.2
PBE/pob-TZVP	6.450	5.448	14.380	505.3
PBE/pob-TZVP-rev2	6.327	5.544	14.590	511.8
B3LYP/pob-TZVP	6.243	5.557	14.375	498.7
B3LYP/pob-TZVP-rev2	6.106	5.637	14.733	507.1
C_5-diamine				
	a, Å	*b*, Å	*c*, Å	*V*, Å^3
Experiment	17.709	6.228	5.857	646.0
Pseudo-experiment	-	-	-	601.3
vdW-DF2/PAW	18.016	6.114	5.972	657.8
PBE/PAW	17.796	6.544	6.071	707.1
PBE/pob-TZVP	17.604	6.071	5.544	592.5
PBE/pob-TZVP-rev2	17.623	6.098	5.567	598.2
B3LYP/pob-TZVP	17.616	5.875	5.647	584.4
B3LYP/pob-TZVP-rev2	17.626	5.915	5.668	590.9
C_6-diamine				
	a, Å	*b*, Å	*c*, Å	*V*, Å^3
Experiment	6.761	5.664	19.003	727.7
Pseudo-experiment	-	-	-	677.4
vdW-DF2/PAW	6.386	5.881	19.702	739.9
PBE/PAW	7.547	5.639	19.970	849.8
PBE/pob-TZVP	6.651	5.347	18.829	669.6
PBE/pob-TZVP-rev2	6.583	5.448	18.942	679.4
B3LYP/pob-TZVP	6.489	5.408	18.757	658.2
B3LYP/pob-TZVP-rev2	6.385	5.553	18.894	669.9
C_7-diamine				
	a, Å	*b*, Å	*c*, Å	*V*, Å^3
Experiment	22.813	6.365	5.751	835.1
Pseudo-experiment	-	-	-	777.3
vdW-DF2/PAW	23.158	6.133	5.964	847.1
PBE/PAW	22.981	7.260	5.743	958.2
PBE/pob-TZVP	22.743	6.296	5.334	763.8
PBE/pob-TZVP-rev2	22.759	6.302	5.385	772.4
B3LYP/pob-TZVP	22.686	5.928	5.596	752.6
B3LYP/pob-TZVP-rev2	22.711	5.996	5.601	762.8
C_8-diamine				
	a, Å	*b*, Å	*c*, Å	*V*, Å^3
Experiment	6.893	5.610	23.527	909.8
Pseudo-experiment	-	-	-	846.9
vdW-DF2/PAW	6.473	5.846	24.384	922.7
PBE/PAW	7.656	5.640	24.900	1075.2
PBE/pob-TZVP	6.728	5.318	23.284	833.1
PBE/pob-TZVP-rev2	6.680	5.416	23.430	847.6
B3LYP/pob-TZVP	6.599	5.344	23.190	817.8
B3LYP/pob-TZVP-rev2	6.524	5.469	23.392	834.7

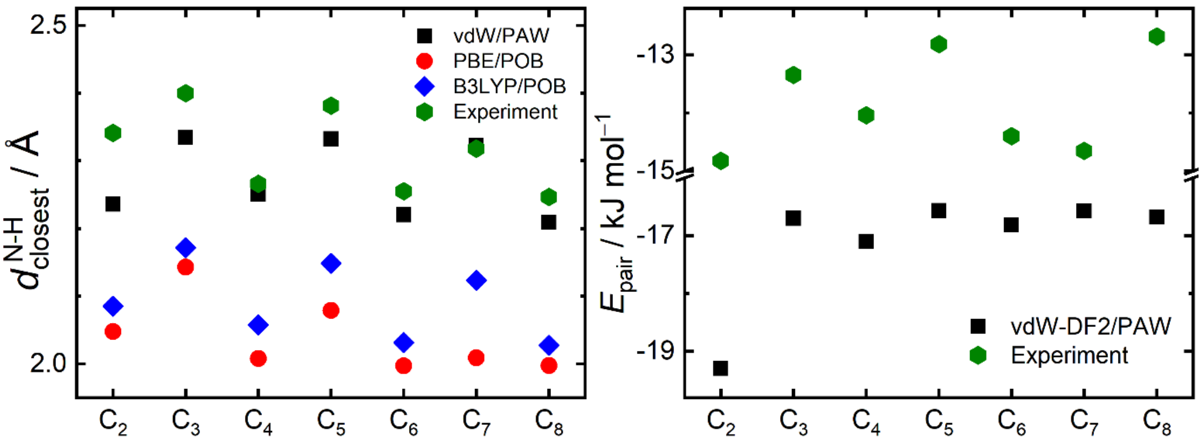

FIGURE S2. Interaction of the nearest-neighbor molecular pairs in crystals of C_n-diamines given by the intermolecular N-H hydrogen bonds extracted from the DFT-optimized or experimental unit-cell geometries: left – the closest contact distances of the N and H atoms; right – MP2/CBS interaction energies of the nearest neighboring molecules. Underestimation of the contact distance by both DFT/pob-TZVP calculations is consistent with the mentioned underestimations of the molar volumes. Concurrent underestimation of the contact distances and overestimation of molar volumes in the vdW-DF2/PAW data set indicates overestimation of the strength of the hydrogen bonding in crystalline C_n-diamines at this level of theory.

TABLE S6

Comparison of the sums of interaction energies of proximate molecular pairs in the crystal lattice $E_{\mathrm{pair}}^{\mathrm{sum}}$ contributing to the cohesive energies (kJ·mol^{-1}) of C$_n$-diamines calculated at various levels of theory. Underlying crystal geometries were previously optimized at the vdW-DF2/PAW level of theory.

	$-E_{\mathrm{pair}}^{\mathrm{sum}}$						
	C_2	C_3	C_4	C_5	C_6	C_7	C_8
Amoeba	81.18	75.54	83.47	89.10	103.47	95.38	119.84
B3LYP-D3/aug-cc-pVTZ	83.18	90.62	99.55	82.73	115.74	89.88	131.95
MP2C-F12/aug-cc-pVTZ	82.87	90.61	98.83	105.91	113.66	120.82	128.79
LCCSD(T)-F12/cc-pVDZ-f12[a]	65.61	77.78	76.98	81.64	86.73	92.33	98.25
MP2/CBS	80.24	87.43	95.76	102.72	110.69	117.78	125.90
CCSD(T)/CBS	80.96	88.24	96.19	103.26	-	-	-

[a] Including local and explicit correlation to the coupled clusters in the LCCSD(T)-F12/cc-pVDZ-f12 method gave $E_{\mathrm{pair}}^{\mathrm{sum}}$ significantly lower in absolute values. Additional calculations of $E_{\mathrm{pair}}^{\mathrm{sum}}$ for C$_2$-diamine indicate that the domain approximations exploiting the projected atomic orbitals omit a significant portion of the correlation energy in our case. Using pair natural orbitals in the locally correlated calculations could improve this data set. Basis set size effect plays also a significant role at this point, unlike the number of orbital pairs treated as strong, which proved to be well converged as all intermolecular orbital pairs were treated as strongly-correlated. Individual $E_{\mathrm{pair}}^{\mathrm{sum}}$ values for C$_2$-diamine calculated with various settings of local and explicit correlation treatment are given in Table S7. Lower-level B3LYP-D3 and Amoeba calculations yielded more scattered $E_{\mathrm{pair}}^{\mathrm{sum}}$, with RMSRs from the linear fits for the series amounting to 6.0 and 13 kJ·mol^{-1}, respectively. The outlying $E_{\mathrm{pair}}^{\mathrm{sum}}$ for the C$_7$-diamine contribute to the high RMSR values significantly. $E_{\mathrm{pair}}^{\mathrm{sum}}$ values calculated by the above-mentioned methods for the experimental geometries are given in Table S8.

TABLE S7
Comparison of the sums of interaction energies of C_2-diamine proximate molecular pairs in the crystal lattice contributing to the cohesive energies (kJ·mol^{-1}) calculated at various levels of theory for the experimental crystal geometries.

	$-E_{coh}$
LCCSD(T)-F12/vdz-f12, 3*A(LOC,FIX)	65.61
LCCSD(T)-F12/vtz-f12, 3*A(LOC,FIX)	71.71
LCCSD(T)-F12/vdz-f12, 3*A(LOC,FIX), all orbital pairs strong	65.26
CCSD(T)-F12/vdz-f12, 3*A(FIX)	77.95
CCSD(T)-F12/vdz-f12, 3*C(HY2,FIX)	76.70
MP2C-F12/avtz, 3*C(HY2,FIX)	82.87
MP2/CBS	80.24
CCSD(T)/CBS	80.96

TABLE S8
Comparison of the sums of interaction energies of proximate molecular pairs in the crystal lattice contributing to the cohesive energies (kJ·mol^{-1}) calculated at various levels of theory for the experimental crystal geometries.

	C_2-diamine	C_3-diamine	C_4-diamine	C_5-diamine	C_6-diamine	C_7-diamine	C_8-diamine
Amoeba	-76.46	-65.83	-82.47	-82.49	-102.78	-97.75	-121.10
B3LYP-D3/aug-cc-pVTZ	-82.06	-85.25	-98.07	-103.69	-112.96	-121.17	-132.47
MP2C-F12/aug-cc-pVTZ	-67.58	-67.85	-78.76	-87.81	-88.98	-91.56	-99.97
LCCSD(T)-F12/cc-pVDZ-f12	-56.35	-55.35	-64.53	-66.17	-71.78	-73.99	-81.36
CCSD(T)/CBS	-68.33	-66.13	-79.49	-82.09	-96.23[a]	-96.42[a]	-93.92[a]

TABLE S9

Long range and many body contributions E_{LRMB} to the cohesive energy (kJ·mol^{-1}) of C$_n$-diamines calculated at the B3LYP-D3/pob-TZVP level of theory for various optimized unit-cell geometries.

	C_2	C_3	C_4	C_5	C_6	C_7	C_8
vdW-DF2/PAW	4.07	5.02	4.97	5.96	4.20	6.28	3.92
PBE-D3/pob-TZVP	4.29	7.15	3.68	9.23	1.58	8.93	1.36
B3LYP-D3/pob-TZVP	5.19	7.57	5.53	10.15	3.20	11.05	2.86

TABLE S10

DFT-calculated cohesive energies E_{coh} (kJ·mol^{-1}) for C$_2$-diamine obtained with varying computational setup used for optimization of the unit-cell geometry (including atomic coordinates) and the following single-point energy calculation. Dispersion correction D3(BJ) was applied for all PBE and B3LYP calculations. POB and POB2 abbreviate the pob-TZVP and pob-TZVP-rev2 basis sets, respectively.

		Single-point energy calculation					
		vdW-DF2/PAW[a]	PBE/PAW[a]	PBE/POB[b]	B3LYP/POB[b]	PBE/POB2[b]	B3LYP/POB2[b,c]
		$-E_{coh}$					
Geometry optimization	Experiment	-73.44	-75.94	-76.66	-80.77	-77.66	-81.49
	vdW-DF2/PAW[a]	-84.20	-83.38	-84.34	-84.67	-85.36	-85.44
	PBE/PAW[a]	-80.15	-84.60	-87.22	-88.18	-88.54	-89.05
	PBE/POB[b]	-80.32	-86.10	-87.29	-88.45	-88.76	-89.33
	B3LYP/POB[b]	-77.29	-83.46	-85.26	-87.42	-86.75	-88.30
	PBE/POB2[b]	-77.61	-84.07	-88.05	-89.06	-89.54	-89.99
	B3LYP/POB2[b]	-77.27	-83.48	-85.92	-87.87	-87.43	-88.97

[a] PAW basis set limited by the 800 eV energy cut-off.

[b] Single-point energies corrected for the BSSE error.

[c] The lowest sensitivity of E_{coh} to the underlying geometry can be seen for both single-point energy refinements using the hybrid functional B3LYP (standard deviation among the six respective E_{coh} values yields 3.0 kJ·mol^{-1}) while the remaining two generalized-gradient approximations showed somewhat higher sensitivity (standard deviation 3.3 – 4.1 kJ·mol^{-1}).

TABLE S11

Comparison of the cohesive energies E_{coh} (kJ·mol^{-1}) of C$_n$-diamines calculated at various levels of theory for the vdW-DF2/PAW-optimized unit-cell and molecular geometries.

	$-E_{coh}$ [a]						
	C_2	C_3	C_4	C_5	C_6	C_7	C_8
vdW-DF2/PAW	80.63	87.71	103.20	111.08	120.29	129.48	142.48
PBE-D3/PAW	75.71	80.49	91.69	95.27	107.10	112.68	124.17
MP2C-F12/aug-cc-pVTZ +B3LYP/POB[a]	68.55	75.32	88.03	94.12	100.00	101.31	121.83
MP2/CBS+Amoeba	69.37	77.20	89.03	95.57	100.26	103.81	122.33
CCSD(T)/CBS+Amoeba	70.09	78.01	89.46	96.11	100.26	-	-

[a] There are overall 24 considered combinations of the short-range E_{pair}^{sum} and long-range E_{LRMB} terms, which were obtained using various computational methods for the vdW-DF2/PAW geometries. Obviously, the PAW calculations tend to overbind the crystals compared to the fragment-based approaches, which can be due to the overestimation of the hydrogen bonding by the DFT/PAW calculations as discussed above.

[b] D3(BJ) correction was included in the B3LYP calculations. POB abbreviates pob-TZVP.

TABLE S12

Minor contributions to the sublimation enthalpy (kJ·mol^{-1}): difference of the zero-point vibration energies (ΔE_{ZPE}), and of the thermal contributions to enthalpy (ΔH_{therm} at 255 K) for the vapor and crystal phase, and the energy term due to the conformer relaxation during sublimation (ΔE^{0}_{conf}).

	C_2-diamine	C_3-diamine	C_4-diamine	C_5-diamine	C_6-diamine	C_7-diamine	C_8-diamine
$E^{\text{lattice a}}_{ZPE}$	1.85	3.60	3.22	2.88	2.70	2.68	2.47
$\Delta E^{\text{A1 b}}_{ZPE}$	-7.29	-8.41	-8.27	-8.31	-9.18	-8.31	-9.56
$\Delta E^{\text{A2 c}}_{ZPE}$	-6.44	-6.67	-8.27	-8.31	-4.86	-	-
$\Delta E^{\text{A3 d}}_{ZPE}$	-7.84	-8.15	-10.22	-10.26	-7.02	-	-
$\Delta E^{\text{A1 b}}_{conf}$	-0.95	0.00	0.00	0.00	0.00	0.00	0.00
$\Delta E^{\text{A2 c}}_{conf}$	-4.46	-5.05	0.00	0.00	-4.55	-	-
$\Delta E^{\text{A3 d}}_{conf}$	-3.71	-3.38	0.00	0.00	-0.30	-	-
$\Delta H^{\text{A1 b}}_{therm}$	0.81	2.55	3.83	2.69	5.12	3.45	7.68
$\Delta H^{\text{A2 c}}_{therm}$	0.73	2.87	3.13	3.51	4.67	-	-
$\Delta H^{\text{A3 d}}_{therm}$	0.62	3.10	2.89	2.08	3.06	-	-

[a] Zero-point energy due to the lattice phonon modes based on the vdW-DF2/PAW quasi-harmonic phonons of the lattice modes.
[b] Simplified model using the vdW-DF2/PAW phonons for crystals and frequencies of the most stable vapor-phase conformer, the DFTBA electronic energies and the sR1SM thermal contributions.
[c] Simplified model using the vdW-DF2/PAW phonons for crystals and frequencies of the most stable vapor-phase conformer, the CCSD(T)/CBS electronic energies and the sR1SM thermal contributions.
[d] Full model using the vdW-DF2/PAW phonons for crystals and scaled B3LYP-D3 frequencies of all vapor-phase conformers, the CCSD(T)/CBS electronic energies, the B3LYP-D3 zero-point energies and the R1SM thermal contributions.

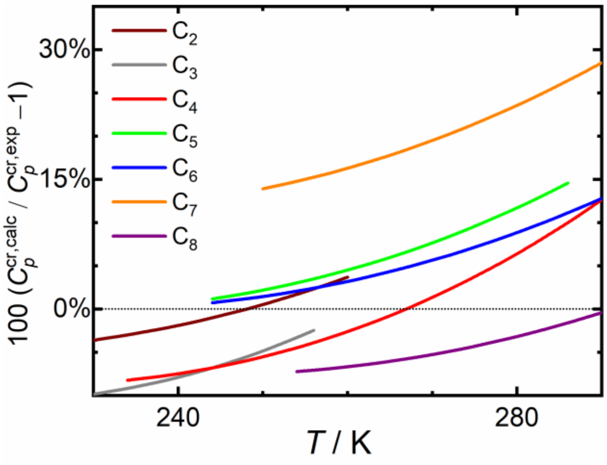

FIGURE S3. Percentage deviations of the quasi-harmonic isobaric heat capacities calculated at the vdW-DF2/PAW level of theory for crystalline diamines.

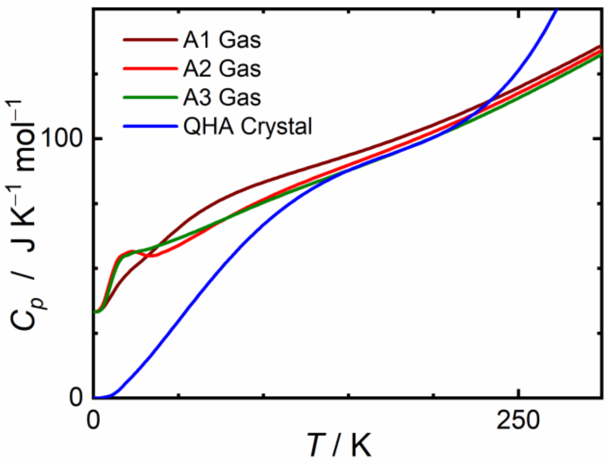

FIGURE S4. Comparison of the isobaric heat capacities C_p of C_4-diamine in the ideal-gaseous state calculated using various conformer-mixing models with the quasi-harmonic C_p of the crystal. Naturally, the three approaches lead to somewhat different C_p^{g0} trends. This happens due to different relative energies, and thus population of individual conformers, especially at very low temperatures.

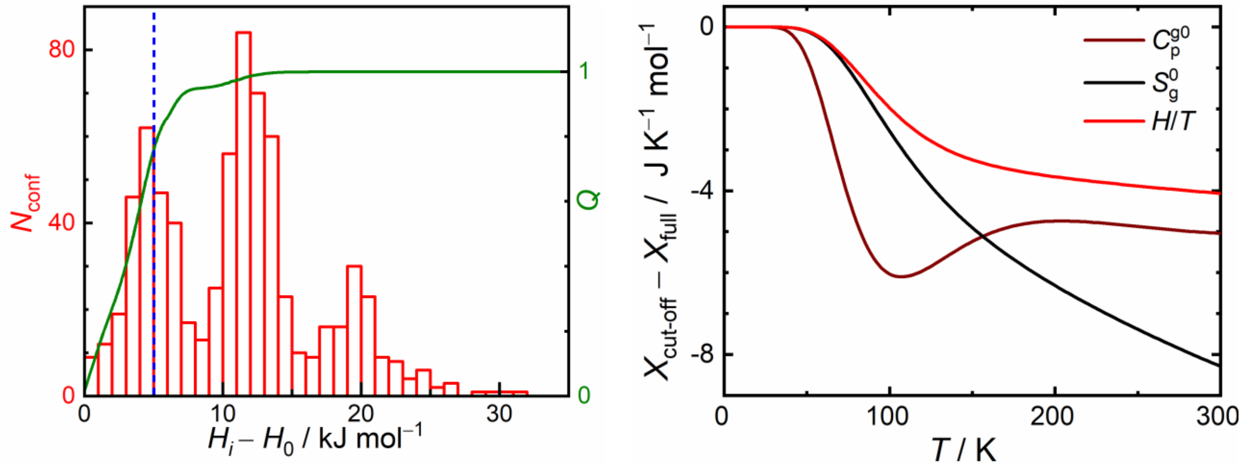

FIGURE S5. Left – histogram of conformer enthalpies of C_6-diamine at 0 K based on CCSD(T)/CBS electronic energies and scaled B3LYP-D3/6-311+G(2df,p) frequencies and a cumulative population Q of the conformers at 298.15 K. Right – absolute errors introduced to the given thermodynamic properties of C_6-diamine in the ideal-gaseous state by omitting all conformers above the 5 kJ mol^{-1} enthalpic cut-off.

TABLE S13

Calculated molar volumes V_m (cm^3 mol^{-1}), isobaric heat capacities C_p^{cr} (J K^{-1} mol^{-1}) and entropies S^{cr} (J K^{-1} mol^{-1}) of crystalline C$_n$-diamines. Isobaric heat capacities C_p^{g0} and entropies S^{g0} of the ideal-gas were obtained using the sR1SM (employing the DFTBA conformer energies, A1 approach)[a] or the R1SM models (A3 approach).

T / K	V_m	C_p^{cr}	S^{cr}	$C_{p,A1}^{g0}$	S_{A1}^{g0}	C_{A3}^{g0}	S_{A3}^{g0}
			C_2-diamine				
0	57.45	0.00	0.00	-	-	-	-
20	57.46	6.42	2.40	37.32	179.19	36.76	179.58
40	57.52	18.73	10.53	40.97	207.05	37.00	205.19
60	57.64	31.27	20.52	44.32	224.67	39.59	220.57
80	57.79	42.46	31.09	48.70	238.24	44.68	232.62
100	57.97	51.77	41.60	53.11	249.71	50.44	243.21
120	58.18	59.17	51.72	57.09	259.81	55.86	252.89
140	58.39	64.95	61.30	60.67	268.91	60.65	261.87
160	58.63	69.59	70.28	64.04	277.24	64.90	270.25
180	58.87	73.71	78.72	67.41	284.97	68.81	278.13
200	59.13	78.04	86.70	70.89	292.24	72.59	285.57
220	59.40	83.40	94.38	74.55	299.15	76.38	292.67
240	59.68	90.67	101.93	78.42	305.79	80.27	299.48
260	59.97	100.79	109.56	82.49	312.21	84.28	306.06
280	60.27	114.74	117.52	86.72	318.45	88.43	312.46
300	60.58	133.56	126.04	91.07	324.57	92.68	318.70

T / K	V_m	C_p^{cr}	S^{cr}	$C_{p,A1}^{g0}$	S_{A1}^{g0}	C_{A3}^{g0}	S_{A3}^{g0}
			C_3-diamine				
0	72.34	0.00	0.00	-	-	-	-
20	72.34	5.18	1.88	49.85	186.50	33.68	186.88
40	72.37	17.90	9.15	51.16	223.47	41.08	211.97
60	72.42	32.50	19.16	54.90	245.51	54.03	231.01
80	72.51	46.25	30.44	59.74	262.26	66.04	248.28
100	72.62	57.80	42.04	64.46	276.26	73.99	263.95
120	72.75	66.70	53.41	68.87	288.50	78.49	277.87
140	72.88	73.11	64.20	73.07	299.49	81.31	290.20
160	73.02	77.67	74.28	77.21	309.55	83.72	301.21
180	73.17	81.39	83.64	81.47	318.91	86.38	311.22
200	73.32	85.56	92.42	85.96	327.74	89.54	320.48
220	73.48	91.77	100.85	90.72	336.17	93.26	329.18
240	73.63	101.78	109.23	95.78	344.28	97.51	337.48
260	73.79	117.56	117.95	101.09	352.16	102.23	345.47

T / K	V_m	C_p^{cr}	S^{cr}	$C_{p,A1}^{g0}$	S_{A1}^{g0}	C_{A3}^{g0}	S_{A3}^{g0}
			C_4-diamine				
0	87.50	0.00	0.00				
20	87.50	6.62	2.44	45.79	190.88	54.93	194.20
40	87.51	21.52	11.38	56.66	228.94	59.19	233.67
60	87.56	38.07	23.24	67.75	255.79	64.22	258.59
80	87.66	53.53	36.35	75.46	277.39	69.89	277.84
100	87.78	66.65	49.76	80.81	295.45	75.45	294.04
120	87.92	77.02	62.87	85.22	311.02	80.59	308.25
140	88.07	84.86	75.36	89.39	324.80	85.44	321.04
160	88.24	90.78	87.09	93.68	337.27	90.26	332.76
180	88.41	95.76	98.08	98.27	348.78	95.26	343.68
200	88.60	101.03	108.43	103.26	359.57	100.61	353.99
220	88.78	108.06	118.37	108.70	369.83	106.35	363.85
240	88.98	118.49	128.19	114.58	379.68	112.50	373.36
260	89.18	134.14	138.25	120.86	389.22	119.02	382.62
280	89.38	156.96	148.97	127.47	398.53	125.85	391.69
300	89.59	189.01	160.83	134.33	407.66	132.90	400.61

C$_5$-diamine							
T / K	V_m	C_p^{cr}	S^{cr}	$C_{p,A1}^{g0}$	S_{A1}^{g0}	C_{A3}^{g0}	S_{A3}^{g0}
0	101.81	0.00	0.00				
20	101.82	8.83	3.28	45.63	198.85	46.17	207.45
40	101.90	26.89	14.75	61.48	238.23	62.41	245.08
60	102.06	46.10	29.30	77.59	268.34	73.39	272.50
80	102.28	63.78	45.04	88.61	293.67	82.75	294.95
100	102.56	78.88	60.95	95.57	315.20	89.85	314.22
120	102.88	91.19	76.46	100.72	333.78	95.45	331.11
140	103.22	101.04	91.28	105.39	350.16	100.53	346.20
160	103.59	109.14	105.32	110.19	364.93	105.67	359.96
180	103.99	116.46	118.60	115.41	378.51	111.20	372.72
200	104.41	124.22	131.26	121.17	391.22	117.27	384.74
220	104.86	133.79	143.53	127.51	403.28	123.91	396.23
240	105.32	146.71	155.69	134.38	414.86	131.11	407.31
260	105.81	164.62	168.10	141.75	426.07	138.78	418.11
280	106.33	189.27	181.16	149.51	437.01	146.84	428.69
300	106.87	222.50	195.29	157.57	447.74	155.19	439.10

C$_6$-diamine							
T / K	V_m	C_p^{cr}	S^{cr}	$C_{p,A1}^{g0}$	S_{A1}^{g0}	C_{A3}^{g0}	S_{A3}^{g0}
0	116.55	0.00	0.00				
20	116.56	10.08	3.75	53.57	206.51	48.84	213.41
40	116.66	29.98	16.65	73.74	251.29	68.98	253.96
60	116.85	50.87	32.78	93.70	285.95	84.99	284.99
80	117.13	70.11	50.11	106.91	315.47	98.05	311.35
100	117.46	86.73	67.59	114.35	340.58	106.17	334.18
120	117.84	100.57	84.67	119.41	362.15	111.50	354.03
140	118.25	111.98	101.06	124.04	381.07	116.20	371.57
160	118.71	121.64	116.65	129.05	398.07	121.26	387.41
180	119.20	130.46	131.49	134.74	413.67	127.06	402.02
200	119.73	139.55	145.70	141.19	428.26	133.68	415.74
220	120.29	150.17	159.48	148.38	442.10	141.11	428.82
240	120.90	163.66	173.10	156.24	455.38	149.26	441.44
260	121.55	181.51	186.87	164.68	468.25	158.03	453.73
280	122.25	205.25	201.15	173.57	480.81	167.29	465.78
300	123.01	236.43	216.31	182.79	493.12	176.90	477.65
320	123.82	275.48	232.46	192.24	505.24	186.76	489.46

C$_7$-diamine							
T / K	V_m	C_p^{cr}	S^{cr}	$C_{p,A1}^{g0}$	S_{A1}^{g0}	C_{A3}^{g0}	S_{A3}^{g0}
0	134.70	0.00	0.00				
20	134.71	11.05	4.09	50.52	212.34		
40	134.83	34.29	18.60	73.94	258.95		
60	135.10	59.37	37.26	99.40	296.68		
80	135.53	82.66	57.60	116.89	329.85		
100	136.10	102.70	78.26	126.38	358.40		
120	136.79	119.21	98.50	132.31	382.91		
140	137.58	132.70	117.92	137.49	404.36		
160	138.49	144.22	136.41	143.06	423.58		
180	139.51	155.25	154.03	149.41	441.18		
200	140.65	167.58	171.00	156.65	457.62		
220	141.94	183.30	187.67	164.76	473.21		
240	143.40	204.70	204.49	173.65	488.16		
260	145.06	234.27	221.98	183.22	502.65		
280	146.97	274.66	240.74	193.32	516.79		
300	149.21	305.73	259.55	203.83	530.66		

C_8-diamine							
T / K	V_m	C_p^{cr}	S^{cr}	$C_{p,A1}^{g0}$	S_{A1}^{g0}	C_{A3}^{g0}	S_{A3}^{g0}
0	145.59	0.00	0.00				
20	145.60	12.26	4.58	54.16	223.88		
40	145.69	35.82	20.10	81.32	275.66		
60	145.87	60.38	39.30	111.28	317.87		
80	146.14	83.16	59.85	131.26	355.15		
100	146.46	103.15	80.61	141.53	387.18		
120	146.82	120.13	100.96	147.70	414.60		
140	147.22	134.33	120.58	153.12	438.52		
160	147.64	146.27	139.31	159.10	459.92		
180	148.09	156.68	157.15	166.06	479.50		
200	148.56	166.38	174.17	174.07	497.78		
220	149.05	176.34	190.49	183.09	515.11		
240	149.56	187.59	206.30	193.01	531.74		
260	150.09	201.22	221.83	203.69	547.87		
280	150.64	218.38	237.35	214.98	563.60		
300	151.21	240.26	253.13	226.72	579.03		
320	151.80	268.08	269.49	238.77	594.23		
340	152.42	303.10	286.75	250.98	609.24		

[a] The simplified models A1 and A2 yield mean absolute deviations (MAD) from the full A3 model equal to 0.9 kJ·mol^{-1} and 0.7 kJ·mol^{-1}.

TABLE S14

Comparison of the experimental sublimation enthalpies $\Delta_{sub}H$ (kJ·mol^{-1}) of C_n-diamines at T = 255 K with the values calculated for the same temperature using the selected levels of theory exhibiting the best performance along with the results of purely periodic calculations, always coupled with the A1 approach for evaluation of the ΔH_{minor} term.

	MAD[a]	RMSR[b]	C_2	C_3	C_4	C_5	C_6	C_7	C_8
Experiment[5]	-	2.1	69.72	79.73	87.38	91.46	104.59	105.61	116.98
CCSD(T)/CBS+B3LYP-D3/POB[c]	1.4%	1.8	70.46	77.36	86.78	91.58	102.43	-	-
MP2/CBS+B3LYP-D3/POB[c]	1.6%	1.8	69.74	76.55	86.35	91.04	102.43	106.64	120.10
MP2C-F12/aug-cc-pVTZ + B3LYP-D3/POB[c]	2.7%	1.7	72.37	79.73	89.42	94.23	105.40	109.68	122.99
PBE-D3/PAW	2.7%	2.4	68.28	74.63	87.25	89.65	103.04	107.82	122.29
vdW-DF2/PAW	12%	2.2	73.20	81.85	98.76	105.46	116.23	124.62	140.60

[a] Mean absolute deviation of the calculated $\Delta_{sub}H$ from the experiment.
[b] Root-mean-square of the residuals of the calculated $\Delta_{sub}H$ from the linear fit of $\Delta_{sub}H$ within the homologous series.
[c] D3(BJ) correction was included in the B3LYP calculations. POB abbreviates pob-TZVP.

TABLE S15

Root-mean-squares of the residuals of calculated $\Delta_{sub}H$ at 255 K from the linear fit within the homologous series. Data were obtained from the given combinations of the short-range and the long-range treatments of the crystal cohesion, vdW-DF2/PAW phonons and frequencies of molecular vibrations, CCSD(T)/CBS calculation of the ΔE^0_{conf} term, and the A1 approach for the vapor phase (sR1SM model based on the DFTBA electronic energies of the conformers).

		Long-range regime treatment			
		Amoeba	HF/pob-TZVP	B3LYP/pob-TZVP	PBE/pob-TZVP
Short-range regime treatment	Amoeba	6.9	7.9	7.7	7.9
	B3LYP-D3/aug-cc-pVTZ	13.5	14.3	14.1	14.2
	MP2C-F12/aug-cc-pVTZ	1.1	1.9	1.7	1.9
	LCCSD(T)-F12/cc-pVDZ-f12	2.7	3.0	2.7	2.6
	CCSD(T)/CBS	1.2	1.9	1.8	1.9
	MP2/CBS	1.2	2.0	1.8	2.0
	Experiment		2.1		

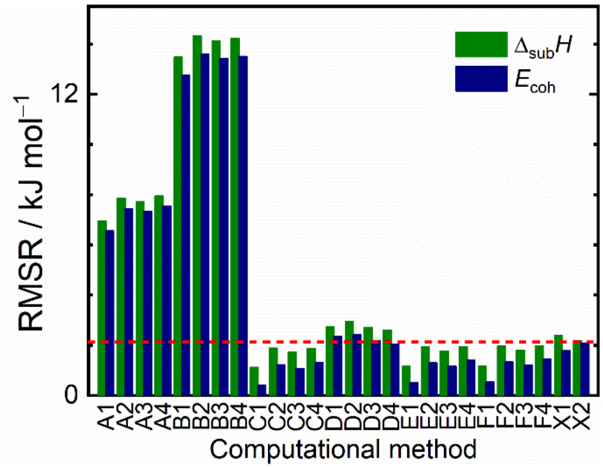

FIGURE S6. Summary of the root-mean-squares of the residuals (RMSR) of the calculated cohesive energies E_{coh} and sublimation enthalpies $\Delta_{sub}H$ at 255 K from the linear fit within the homologous series. Short-range treatments: A – Ameoba; B – B3LYP-D3/aug-cc-pVTZ; C – MP2C/aug-cc-pVTZ; D – LCCSD(T)-F12/cc-pVDZ-f12; E – CCSD(T)/CBS; F – MP2/CBS. Long-range treatments: 1 – Amoeba; 2 – HF/pob-TZVP; 3 – B3LYP/pob-TZVP; 4 – PBE/pob-TZVP. X1 – PBE-D3(BJ)/PAW; X2 – vdW-DF2/PAW. Red dashed line shows the RMSR for the experimental data.

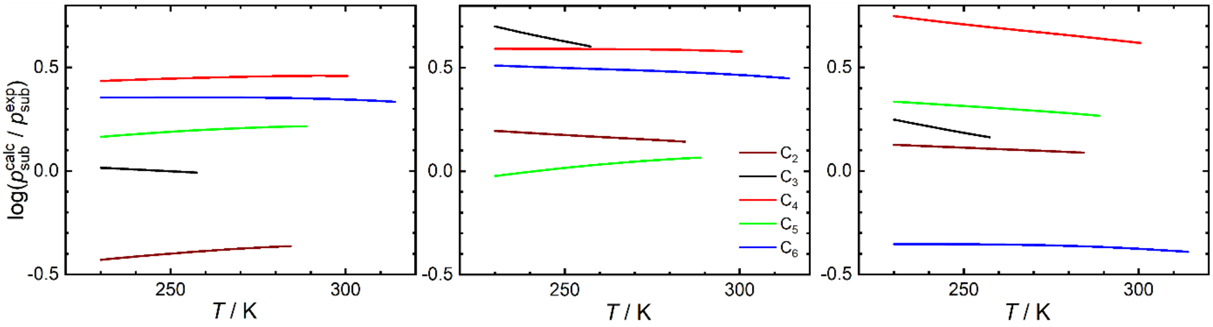

FIGURE S7. Logarithmic deviations of the calculated sublimation pressures from the experimental data. MP2/CBS+HF/pob-TZVP cohesive energies are combined with various treatments of the vibration and conformation-based terms: left – A1; middle – A2; right – A3. Substituting the A1 approach with its more sophisticate alternatives (A2, A3) does bring neither an improvement of accuracy, nor any systematic trend for deviations of temperature-dependent p_{sub} from the experimental values.
